# Dual RNA-Seq Analysis of *Trichophyton rubrum* and HaCat Keratinocyte Co-Culture Highlights Important Genes for Fungal-Host Interaction

**DOI:** 10.3390/genes9070362

**Published:** 2018-07-19

**Authors:** Monise Fazolin Petrucelli, Kamila Peronni, Pablo Rodrigo Sanches, Tatiana Takahasi Komoto, Josie Budag Matsuda, Wilson Araújo da Silva, Rene Oliveira Beleboni, Nilce Maria Martinez-Rossi, Mozart Marins, Ana Lúcia Fachin

**Affiliations:** 1 Biotechnology Unit, University of Ribeirão Preto-UNAERP, São Paulo 2201, Brazil; mofazolin@gmail.com (M.F.P.); tattytk@hotmail.com (T.T.K.); josie@unidavi.edu.br (J.B.M.); rbeleboni@unaerp.br (R.O.B.); mmarins@gmb.bio.br (M.M.); 2 Laboratory of Molecular Genetics and Bioinformatics, Regional Hemotherapy Center of Ribeirão Preto, Ribeirão Preto 2501, Brazil; kcperoni@gmail.com (K.P.); wilsonjr@usp.br (W.A.d.S.J.); 3 Department of Genetics, Ribeirão Preto Medical School, University of São Paulo, Ribeirão Preto 14049-900, Brazil; psanches@gmail.com (P.R.S.); nmmrossi@usp.br (N.M.M.-R.); 4 Center for Medical Genomics at the Clinics Hospital of Ribeirão Preto Medical School, University of São Paulo, Ribeirão Preto 14049-900, Brazil

**Keywords:** dermatophytes, *ERG6*, epithelial barrier, glyoxylate cycle, fungal-host interaction

## Abstract

The dermatophyte *Trichophyton rubrum* is the major fungal pathogen of skin, hair, and nails that uses keratinized substrates as the primary nutrients during infection. Few strategies are available that permit a better understanding of the molecular mechanisms involved in the interaction of *T. rubrum* with the host because of the limitations of models mimicking this interaction. Dual RNA-seq is a powerful tool to unravel this complex interaction since it enables simultaneous evaluation of the transcriptome of two organisms. Using this technology in an in vitro model of co-culture, this study evaluated the transcriptional profile of genes involved in fungus-host interactions in 24 h. Our data demonstrated the induction of glyoxylate cycle genes, *ERG6* and *TERG_00916*, which encodes a carboxylic acid transporter that may improve the assimilation of nutrients and fungal survival in the host. Furthermore, genes encoding keratinolytic proteases were also induced. In human keratinocytes (HaCat) cells, the *SLC11A1*, *RNASE7*, and *CSF2* genes were induced and the products of these genes are known to have antimicrobial activity. In addition, the *FLG* and *KRT1* genes involved in the epithelial barrier integrity were inhibited. This analysis showed the modulation of important genes involved in *T. rubrum*–host interaction, which could represent potential antifungal targets for the treatment of dermatophytoses.

## 1. Introduction

Dermatophytoses are superficial infections of keratinized tissues caused by a group of filamentous fungi called dermatophytes [1]. Although these infections are restricted to the superficial layers of the epidermis, they can become invasive and can lead to severe diseases in immunocompromised [2] and diabetic patients [3]. Data from the World Health Organization estimate that approximately 25% of the world’s population have skin infections caused by fungi.

Most human dermatophytoses are caused by anthropophilic dermatophytes. Among these species, *Trichophyton rubrum* is the main cause of dermatophytoses in the world [4,5]. It is estimated that *T. rubrum* is the etiological agent of 69.5% of all cases of dermatophytosis caused by species of the genus *Trichophyton*, followed by *Trichophyton interdigitale, Trichophyton verrucosum* and *Trichophyton tonsurans* [6].

Despite the importance of these infections in clinical practice, knowledge of the molecular mechanisms involved in the dermatophyte-host interaction is limited, possibly because of the technical difficulties of the models mimicking this interaction, as well as the lack of genetic tools that allow for a more in-depth study of these organisms [7]. However, this scenario has been changing with the sequencing of mixed transcriptomes, also called dual RNA-seq, an approach widely used for the study of the complex interaction that exists between the host and pathogen [8] including bacteria [9], viruses [10], and fungi [11,12].

With the advent of this technology and the published sequence of the *T. rubrum* genome, the present study evaluated the transcriptional profile of *T. rubrum* co-cultured with human keratinocytes (HaCat) for 24 h by dual RNA-seq to identify important genes involved in the host defense and fungal pathogenicity in order to increase our understanding of the molecular aspects of this interaction. After 24 h of co-culture, we observed the induction of specific genes of the glyoxylate cycle and of a carboxylic acid transporter in *T. rubrum*, which may contribute to metabolic flexibility in nutrient-limited host niches, as well as of the *ERG6* gene involved in plasma membrane permeability, which may favor the assimilation of nutrients and fungal survival in the host. In addition, we found that the modulation of the *LAP2* and *DPPV* genes involved in the production of keratinolytic proteases that are important for the virulence of this dermatophyte. In contrast, in keratinocytes, genes involved in the repair of the epithelial barrier, in the increase of cell migration and the *RNASE7*, *SLC11A1* and *CSF2* genes (whose gene products have potential antimicrobial activity) were induced. Furthermore, the inhibition of *FLG* and *KRT1* genes whose products are directly involved in the maintenance of skin barrier integrity was observed.

## 2. Materials and Methods

### 2.1. Strains, Media and Growth Conditions

The *T. rubrum* strain CBS 118892 (CBS-KNAW Fungal Biodiversity Center, Utrech, The Netherlands) sequenced by the Broad Institute (Cambridge, MA, USA) was cultured on Sabouraud dextrose agar (Oxoid, Hampshire, UK) for 15 days at 28 °C.

### 2.2. Keratinocytes, Media and Growth Conditions

The immortalized human keratinocytes cell line HaCat was purchased from Cell Lines Service GmbH (Eppelheim, Germany). The cells were cultured in an RPMI medium (Sigma Aldrich, St. Louis, MO, USA) supplemented with 10% fetal bovine serum at 37 °C in a humidified atmosphere containing 5% CO_2_. Antibiotics (100 U/mL penicillin and 100 μg/mL streptomycin) were added to the medium to prevent bacterial contamination.

### 2.3. Co-Culture Assay and Conditions

For co-culture assay, a ratio of 2.5 × 10^5^ cells/mL of keratinocytes to 1 × 10^7^ conidia/mL of *T. rubrum* solution was used, and the co-culture was performed as described in [13]. The assays were carried out in three independent experiments performed in triplicate. Cultured keratinocytes and *T. rubrum* conidia were used as controls and were cultured similarly to the co-infection in RPMI Medium (Sigma Aldrich). Scanning electron microscopy was performed with a JEOL JEM 100CXII electron microscope at the Multiuser Electron Microscopy Laboratory of the Department of Cell and Molecular Biology (Ribeirão Preto Medical School, São Paulo, Brazil) to determine whether the penetration of fungal hyphae into keratinocytes occurred within 24 h of co-culture. The cell viability of HaCat keratinocytes prior to *T. rubrum* inoculation and after 24 h of co-culture was determined by measuring the release of the enzyme lactate dehydrogenase (LDH) (TOX7 kit from Sigma-Aldrich) in the RPMI Medium (Sigma Aldrich) according to the manufacturer’s instructions and described in [14]. The absorbance was read in a microplate reader (Elx 800 UV Bio-Tek Instruments, Inc., Winooski, VT, USA) at 490 nm.

### 2.4. RNA Isolation and Integrity Analysis

After 24 h of incubation, fungi and human cells were recovered by scraping and centrifuging at 1730× *g* for 10 min. For the disruption of the fungal cell wall, the samples (co-culture and controls) were treated with lysis solution (20 mg/mL of lysing enzymes from *Trichoderma harzianum* purchased from Sigma-Aldrich; 0.7 M KCl and 1 M MgSO_4_, pH 6.8) for 1 h at 28 °C under gentle shaking, followed by centrifugation at 1000× *g* for 10 min, as described in [13]. Total RNA was extracted using the Illustra RNAspin Mini RNA Isolation Kit (GE Healthcare, Chicago, IL, USA) according to the manufacturer’s instructions. After extraction, the absence of proteins and phenol in the RNA was analyzed in a MidSci Nanophotometer (Midwest Scientific, St. Louis, MO, USA) and the RNA integrity was assessed by microfluidic electrophoresis in an Agilent 2100 Bioanalyzer (Agilent Technologies, Santa Clara, CA, USA). Only RNA with an RNA integrity number (RIN) >7.0 was used. These RNAs were quantified in a Quantus™ Fluorometer (Promega Corporation, Madison, WI, USA) to verify if they had the adequate concentration for library construction.

### 2.5. Library Construction and Sequencing

The cDNA libraries for RNA sequencing were constructed in triplicate for each condition (cultured keratinocytes and *T. rubrum* conidia as control and co-culture). The libraries were constructed using the TrueSeq^®^ RNA Sample Preparation Kit v2 (Illumina, San Diego, CA, USA) according to manufacturer’s instructions and the libraries were validated according to the Library quantitative PCR (qPCR) Quantification Guide (Illumina). A pool of 11 pM of each library was distributed on the flowcell lanes and cluster amplification was performed in a cBot (Illumina) according to the manufacturer’s instructions.

Single read and paired-end sequencing were performed in a Genome Analyzer IIx and Hiseq 2000 (Illumina), respectively, according to the manufacturer’s instructions. The RNA-seq data are deposited in the GEO (Gene Expression Omnibus) database [15] under the accession number GSE110073

### 2.6. Sequence Data Analysis

The reads generated for each library were filtered using the FastQC software (https://www.bioinformatics.babraham.ac.uk/index.html) for removal of Illumina adapters and poor-quality reads. Only those with a Phred score > 20 were considered high-quality reads.

The high-quality reads were aligned to the *T. rubrum* reference genome of the Broad Institute’s Dermatophyte Comparative Database and to the *Homo sapiens* reference genome HG19 [16].

After alignment, the triplicate of each library was normalized according to each library size and the number of reads was calculated using the summarize Overlaps function in the Genomic Ranges Bioconductor package, obtaining the expression levels of the transcripts in the samples. For statistical evaluation of the gene expression data between the samples, the false discovery rate (FDR) procedure was applied using the DEseq package [17] implemented in the R/Bioconductor software. Genes exhibiting statistical significance <0.05 and a log_2_ fold change ratio ≥1 or ≤−1 were defined as differentially expressed genes (DEGs). The functional categorization of *T. rubrum* and keratinocyte DEGs in co-culture was performed according to Gene Ontology [18] using the Blast2GO algorithm [19] for *T. rubrum* and the website http://www.geneontology.org/ for human keratinocyte DEGs. For functional enrichment, the BayGO algorithm [20] and Enrichr enrichment tool [21,22], were used for the *T. rubrum* and keratinocyte DEGs, respectively. A *p*-value < 0.05 indicated the over-represented categories.

### 2.7. qPCR Validation

A set of 14 genes, including the *T. rubrum* and keratinocyte genes, were selected for validation by qPCR. For the reaction, 1 μg of the total RNA used for sequencing was treated with DNAse 1 Amplification Grade^®^ (Sigma Aldrich) to remove any genomic DNA contamination. The High-Capacity cDNA Reverse Transcription^®^ Kit (Applied Biosystems, Foster city, CA, USA) was used for cDNA conversion according to the manufacturer’s instructions. Quantitative Real Time (RT)-PCR experiments were performed in triplicate using the SYBR Taq Ready Mix Kit (Sigma Aldrich) in a Mx3300 qPCR System (Stratagene, San Diego, CA, USA). The cycling conditions were initial denaturation at 94 °C for 10 min, followed by 40 cycles at 94 °C for 2 min, at 60 °C for 60 s and at 72 °C for 1 min. A dissociation curve was constructed at the end of each PCR cycle to verify single product amplification. Gene expression levels were calculated using the 2^−ΔΔ*C*^_T_ comparative method. GAPDH [23] and β-actin [24] were used as normalizer genes for keratinocytes and 18S [25] and β-tubulin [26] as normalizer genes for *T. rubrum*. The results are reported as the mean ± standard deviation of three experiments. Pearson’s correlation test was used to evaluate the correlation between the qPCR and RNA-seq techniques. The primers used for qPCR validation are available in Table S4.

## 3. Results

### 3.1. Electron Microscopy of T. rubrum and HaCat Co-Culture

**Figure 1:**
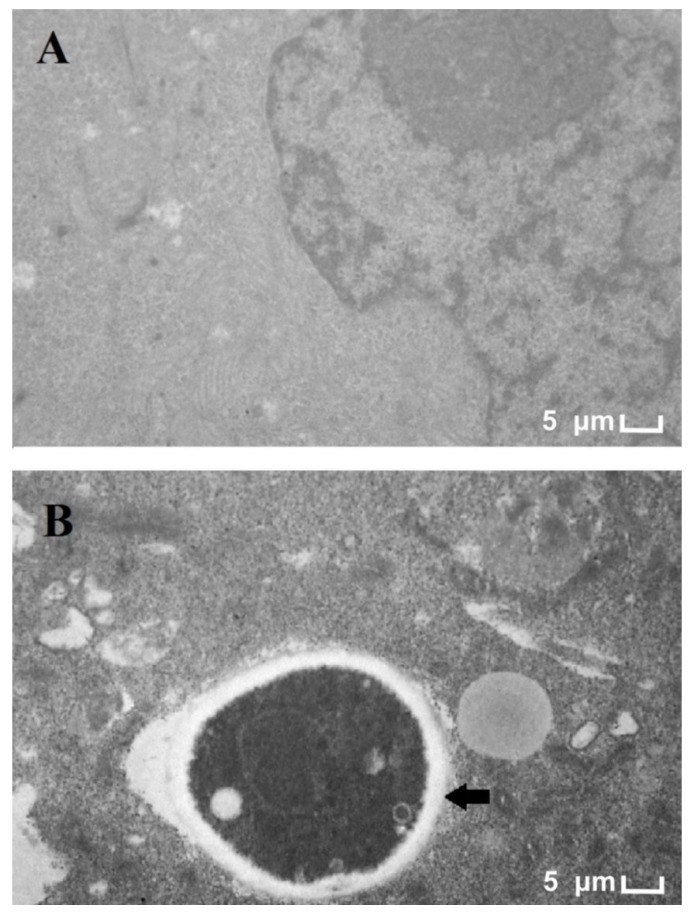
The transmission electron microscopy of the *Trichophyton rubrum*-HaCat co-culture after 24 h. (**A**) Human keratinocytes (HaCat) keratinocyte as the control (14kx); (**B**) Co-culture (14kx). The arrow indicates a fragment of *T. rubrum* hyphae inside the HaCat cells.

Figure 1B shows the penetration of a *T. rubrum* hypha into a HaCat cell after 24 h of co-culture. Thus, the period of co-culture was considered appropriate for the evaluation of the fungal-host interaction.

We performed the LDH assay with 24 h of co-culture to evaluate the keratinocyte cell viability. The percentage of LDH release was 18%. This LDH release may be due to the penetration of some fungal hyphae into keratinocyte cells (as observed in Figure 1B). LDH release was also evaluated at 0 h to assess cell viability prior to the addition of the fungus. The LDH release rate at 0 h was 1%. As a positive control, Triton X-100 (1%) was used in which 100% of the LDH release was obtained. Considering that we used 2.5 × 10^5^ cells/mL prior to inoculation of the fungus and that the percentage of LDH was 18%, we can estimate that approximately 2 × 10^5^ cells/mL are still viable in 24 h of co-cultivation.

### 3.2. Dual RNA-Seq Analysis of the Fungal-Host Interaction

Sequencing resulted in an average of 40, 34 and 47 million raw reads corresponding to the libraries of *T. rubrum* conidia, co-culture, and keratinocytes, respectively. Low-quality reads were then removed, and the resulting reads were aligned to the references genomes of *T. rubrum* and *Homo sapiens* HG19 (UCSC Genome Bioinformatics site, Santa Cruz, CA, USA). On average, 85% and 5% of the quality reads of the *T. rubrum* conidia and co-culture libraries, respectively, aligned to the *T. rubrum* reference genome (CBS 118892). These percentages were 84% and 85%, respectively, when the quality reads of the co-culture and keratinocyte cell line were aligned to the HG19 reference genome. The total number of filtered and aligned reads of each library is shown in Table S1.

### 3.3. Transcriptional Profile Analysis of Differentially Expressed Genes in the T. rubrum-Keratinocyte Co-Culture System

Table 1 and Table 2 show the genes that are up-regulated and down-regulated in keratinocytes and *T. rubrum*, respectively. According to the distribution of the genes, those showing a *p*-value < 0.05 and log_2_ fold change ≥1 or ≥−1 in each condition were considered differentially expressed (Figure S1). A total of 353 HaCat genes and 70 *T. rubrum* genes were differentially expressed during 24 h of co-culture (Tables S2 and S3). 

### 3.4. Functional Categorization of Differentially Expressed Genes

**Figure 2:**
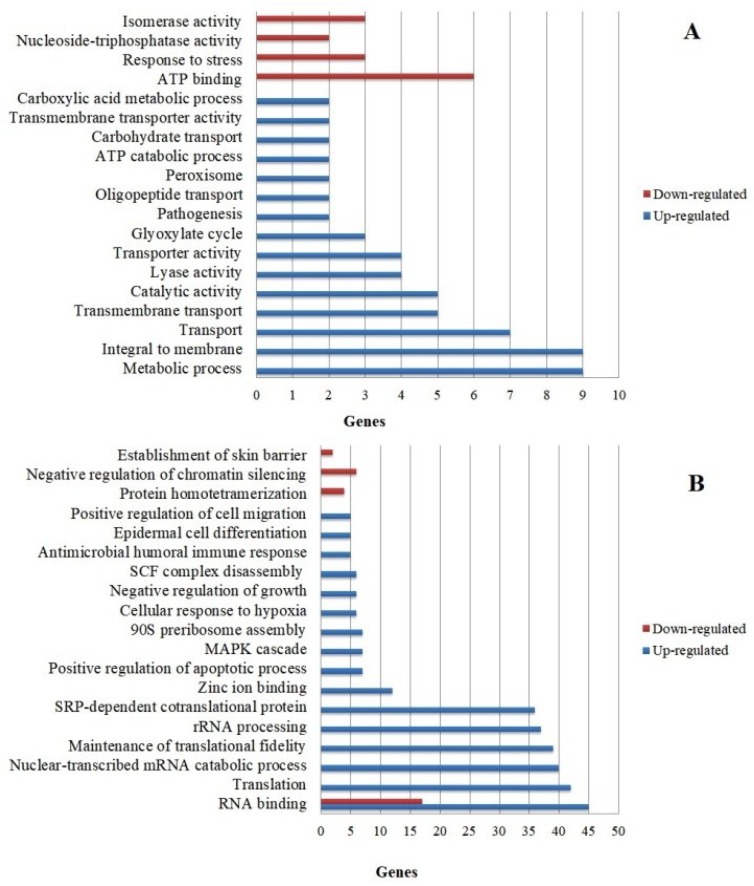
The gene Ontology-based functional categorization of differentially expressed genes. The main representative functional categories (*p* < 0.05) of genes differentially expressed in *T. rubrum* (**A**) and HaCat (**B**).

To evaluate the molecular and biological mechanisms involved in the fungal-host interaction, the DEGs were categorized according to biological processes and molecular functions. The most enriched categories considering a *p* < 0.05 are shown in Figure 2.

Most of the up-regulated *T. rubrum* genes (Figure 2A) belong to categories related to metabolic processes, membrane proteins, and substance transport, while the down-regulated genes are mainly involved in ATP binding. However, categories important to the fungus-host relationship, such as those including genes involved in the glyoxylate cycle and pathogenicity, should also be highlighted. Table 3 shows some functional categories that are important for the interaction of *T. rubrum* with HaCat keratinocytes. Within these categories, we selected some genes considered to play a fundamental role in the attack mechanisms and survival of the fungus when in contact with the host for validation and discussion: genes involved in protease secretion (*TERG_12606; TERG_08405*), metabolic flexibility for nutrient assimilation (*TERG_01281*; *TERG_11638*; *TERG_11639*; *TERG_00916*), and plasma membrane permeability (*TERG_03102*). On the other hand, up-regulated genes in keratinocytes (Figure 2B) are mainly found in the categories related to RNA binding, translation, and rRNA processing, while most of the down-regulated genes belong to the RNA binding category. Furthermore, Table 4 shows some functional categories that are important for the cell defense mechanisms of human keratinocytes during co-culture with *T. rubrum*, such as the genes involved in the innate immune response, epidermal cell differentiation, regulation of cell migration, and establishment of the skin barrier.

### 3.5. Validation by qPCR

**Figure 3:**
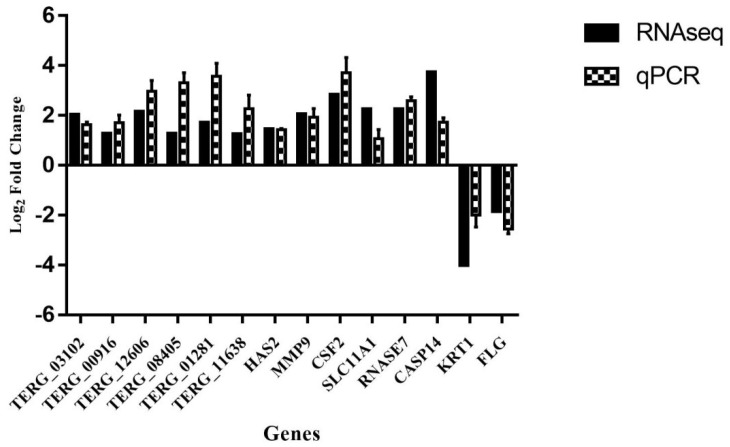
The comparison of gene modulation obtained by RNA-seq and quantitative PCR (qPCR)**.** The error bars represent the standard error of three independent replicates. Pearson’s test indicated a strong correlation between the two techniques (r = 0.80; *p* < 0.01).

Pearson’s correlation test was used to evaluate the correlation between dual RNA-seq and qPCR. For this purpose, 14 genes were chosen for validation, including 6 *T. rubrum* genes (*TERG_11638*; *TERG_01281*; *TERG_08405*; *TERG_12606*; *TERG_00916*; *TERG_03102*) and 8 HaCat genes (*HAS2*; *CSF2*; *SLC11A1*; *RNASE7*; *CASP14*; *MMP9*; *KRT1*; *FLG*). Figure 3 shows the comparison of the log_2_ fold change values obtained with the two techniques. The gene expression results obtained by RNA-seq showed a strong correlation (r = 0.80, *p* < 0.001) with the gene modulation values obtained by qPCR. This finding suggests that sequencing provided reliable results, demonstrating the reproducibility and accuracy of the technique.

## 4. Discussion

Through the analysis of mixed transcriptomes, this is the first study to sequence by dual RNA-seq the dermatophyte *T. rubrum* with a HaCat cells in an in vitro model of co-culture for 24 h.

Based on the sequencing data generated, only about 5% of the quality reads of the co-culture could be aligned to the *T. rubrum* reference genome (CBS 118892), indicating a predominance of human reads in this library. Indeed, a major challenge encountered in the sequencing of mixed transcriptomes is the difference in the amount of RNA between different cell types. Whereas a human cell contains about 20–25 pg RNA, a fungal cell contains 0.5–1 pg [8,27], a fact that may explain the smaller number of reads generated for *T. rubrum* compared to human keratinocytes. This obstacle was also observed in dual RNA-seq analysis of a *Magnaporthe oryzae* and *Oryza sativa* co-culture, in which the percentage of alignment of fungal reads to the *M. oryzae* reference genome ranged from 0.1–0.2% [28]. As the latest example, in [12] also obtained a low percentage (~1%) of reads corresponding to the pathogen *Phytophthora cinnamomi* in dual RNAseq with *Eucalyptus nitens*. In that study, the authors obtained 283 genes of *Phytophthora cinnamomi* in a genome comprising approximately 58.38 Mb (National Center for Biotechnology). Comparing these data with our study, we obtained about 5% of read alignment and 70 modulated *T. rubrum* genes considering a fold change ≥1 or ≤−1 within a genome of 22.5 Mb.

However, we reached coverage of 90.7% of the 8.616 annotated genes in the *T. rubrum* genome considering the genes with at least one count read.

Seventy DEGs of *T. rubrum* were identified after 24 h of co-culture, which could be allocated to different categories according to biological function. Categories that were relevant for the understanding of the attack mechanisms of *T. rubrum* against keratinocytes included those containing *TERG_12606* and *TERG_08405* which encode important proteases for tissue invasion by the fungus, *TERG_03102* or the *ERG6* gene which is considered a promising target for the development of new antifungal agents [29], and *TERG_01281*, *TERG_11638* and *TERG_ 00916* which may be involved in the metabolic flexibility of *T. rubrum*, improving the adaptation and development in the host.

Regarding the functional categories containing the 353 HaCat DEGs, we highlight the following genes as important for the host defense mechanisms: *SLC11A1*, *RNASE7* and *CSF2* involved in innate immune response signaling; *MMP9* and *HAS2* involved in the regulation of epithelial cell migration; *KRT1* and *FLG* involved in maintaining skin barrier integrity, and *CASP14* involved in epidermal cells differentiation.

### 4.1. Genes Involved in Protease Secretion Are Important for the Pathogenicity of T. rubrum

During the course of infection, dermatophytes such as *T. rubrum* secrete endo- and exoproteases that degrade the keratin of the host tissue into oligopeptides and amino acids [30]. These compounds are used as a source of carbon, nitrogen, phosphorus, and sulfur for nutrition of the fungus [31].

The results of dual RNA-seq showed the induction of *TERG_12606* (log_2_ fold change: 2.16) and *TERG_08405* (log_2_ fold change: 1.29) (functional category: pathogenicity), which encode exoproteases (dipeptidyl peptidase V and leucine aminopeptidase 2, respectively). These findings corroborate the results of Reference [32] which evaluated gene expression by microarray in *T. rubrum* grown in a keratin-containing medium, and in [33] which evaluated the secretion of exoproteases, including dipeptidyl peptidase V, by *T. rubrum* in a keratin-containing medium. The secretion of endo- and exo-proteases by dermatophytes is one of the best-characterized virulence factors [32,33] and is of fundamental importance for invasion and dissemination of the fungus through the stratum corneum of the host [34].

### 4.2. The *ERG6* Gene Is a Promising Target for Developing a New Antifungal Agent Against T. rubrum

In addition to the need of effective degradation of skin protein components for penetration of the fungus into tissue, the maintenance of fungal plasma membrane permeability and fluidity is essential for the correct assimilation of nutrients and the consequent growth and survival of *T. rubrum* in the host. In the present study, we observed the induction of *TERG_03102* (log_2_ fold change: 2.05) (functional category: metabolic process), which corresponds to the *ERG6* gene. This gene encodes the enzyme 24-C-methyltransferase, which participates in ergosterol biosynthesis [35]. Ergosterol is known to be responsible for fungal plasma membrane fluidity and permeability and it is important for the adequate function of membrane-anchored proteins [36].

The ergosterol biosynthesis pathway, which is absent in mammals, is the target of antifungal agents such as terbinafine. However, new genes of this pathway should be explored as potential targets because of reports of resistance of *T. rubrum* to this commercial antifungal drug [37]. One example of a promising potential target of new antifungals is the *ERG6* gene whose expression was found to be modulated in this study. Altered expression of this gene results in plasma membrane changes, impairing the transport of nutrients into the fungal cell [38]. The importance of this gene as a new therapeutic strategy has also been reported in [29]. In a comparative genomics study, these authors identified this gene in important human fungal pathogens such as *Candida albicans* and *Aspergillus fumigatus*.

### 4.3. Glyoxylate Cycle Genes and a Carboxylic Acid Transporter May Be Associated with Mechanisms of Metabolic Flexibility in the T. rubrum-Host Relationship

Additionally, regarding the importance of nutrient assimilation by the fungus for its development during infection, the metabolic flexibility of some pathogenic fungi is worth noting. This flexibility enables the fungus to obtain nutrients through the assimilation of alternative carbon sources in nutrient-limited host niches [39,40]. Knowledge of the genes that are induced to favor this metabolic flexibility is still limited. Thus, these genes are interesting targets for the development of more selective antifungals since the induction of alternative metabolic pathways is an exclusive property of pathogenic fungi [41].

In the present study, genes involved in metabolic flexibility were modulated: *TERG_01281* (log_2_ fold change: 1.72), *TERG_11638* (log_2_ fold change: 1.26) and *TERG_11639* (log_2_ fold change: 1.13) (functional category: glyoxylate cycle), which encode malate synthase and isocitrate lyases, respectively, are enzymes that participate in the glyoxylate cycle. In other clinical fungi, the activation of this cycle permits cell survival in low-glucose environments through the synthesis of glucose from lipids and other carbon sources [41]. We suggest this strategy could favor the growth and persistence of *T. rubrum* in the host since the fungus infects tissues rich in keratin and lipids. Furthermore, this cycle provides pathogenicity and virulence to other pathogens such as *C. albicans*, since the alternative assimilation of nutrients in nutrient-limited host niches favors pathogen survival and adaptation to the host [39].

The role of the glyoxylate cycle in the pathogenicity of *T. rubrum* is still not well established considering that this fungus causes superficial infections. However, we also showed the induction of genes encoding isocitrate lyase and malate synthase during the co-culture of HaCat keratinocytes with *T. rubrum.* The same genes were repressed in the presence of antifungal compounds licochalcone and caffeic acid in the co-culture for 24 h [42]. We also highlight the induction of *TERG_00916* (log_2_ fold change: 1.28), which encodes a carboxylic acid transporter (functional category: transport), and suggest that the fungus can use this transporter to facilitate the assimilation of carboxylic acids as an alternative carbon source during infection. The expression of two short-chain carboxylic acid transporters has been demonstrated in *C. albicans* when glucose availability in the host is low. These findings indicate the importance of these transporters in the early stages of infection, contributing to the virulence of the pathogen [43].

### 4.4. The Modulation of Genes Involved in the Maintenance of the Skin Barrier, Cell Migration, and Differentiation May Be Associated with the Defense Strategies of Human Keratinocytes

The degradation of keratin present in the epidermis through the secretion of proteases such as those modulated in this study (*TERG_12606* and *TERG_08405*) causes marked changes in the function and structure of the epithelial barrier [44]. Repression of the *FLG* (log_2_ fold change: −1.86) and *KRT1* (log_2_ fold change: −4.02) genes that encode filaggrin and keratin 1, respectively, was observed during the 24 h of co-culture of *T. rubrum* with HaCat cells. We suggest the repression of the *FLG* and *KRT1* genes to be related to the loss of skin barrier integrity, favoring the installation and tissue invasion by the fungus since the proteins encoded by these genes act together during the transition of keratinocytes to corneocytes that will compose the epithelial barrier [45,46]. These results corroborate the findings reported in [47] which identified the reduced expression of filaggrin in cases of tinea corporis caused by *T. rubrum*, and in [48] which observed the loss of skin barrier integrity in *KRT1*-deficient mice.

In the case of damage to the skin barrier, creating a portal of entry for exogenous microorganisms, epithelial cells respond rapidly to close the wound by increasing cell proliferation. In addition, the remodeling of affected tissue occurs and the migration of epithelial and immunocompetent cells to the site of infection is facilitated [49,50].

Among the genes allocated to the functional category of epidermal cell differentiation, the most modulated gene was *CASP14* (log_2_ fold change: 3.74), which encodes caspase 14 (Table 4). This is the only caspase not involved in apoptotic pathways [51,52] and an increase in its expression is associated with the differentiation of keratinocytes into corneocytes [53,54], demonstrating a low accumulation of filaggrin fragments in the stratum corneum and increased epithelial water loss in caspase 14-deficient mice. Thus, the induction of *CASP14* expression might be related to the increased differentiation of keratinocytes into corneocytes in an attempt to strengthen the epithelial barrier. Another possibility is that the increased expression of the *CASP14* gene is involved in the repair of damage caused by the repression of the *FLG* and *KRT1* genes as a host defense response during infection with *T. rubrum*.

Regarding the functional category containing genes involved in the regulation of cell migration, the induction of the *MMP9* gene (log_2_ fold change: 1.46), which encodes matrix metalloproteinase 9, should be highlighted (Table 4). In addition to the role of matrix metalloproteinases in the remodeling of damaged tissues through the degradation of extracellular matrix, studies have shown that matrix metalloproteinase 9 is necessary for the migration of inflammatory cells to the epidermis [55]. Considering the data available so far, the induction of this gene may indicate an important role in the regulation of the flow of immunocompetent cells through the epidermal compartment in infections caused by *T. rubrum*. Since this protein is produced in its inactive form [56], the present results do not permit to establish whether the matrix metalloproteinase 9 becomes active in keratinocytes during dermatophyte infections. Furthermore, the increased expression of this enzyme in its active form may be associated with an increase in inflammation and the occurrence of ulcers in some diseases such as ocular herpes [57] and leishmaniasis [58], in addition to facilitating the dissemination of the pathogen through tissues by excessive cleavage of collagen IV present in the basement membrane [59].

With respect to other genes involved in the regulation of cell migration, the induction of the *HAS2* gene was observed (log_2_ fold change: 1.46), which encodes hyaluronan synthase 2, an enzyme that participates in the synthesis of hyaluronic acid. This acid is one of the main components of the extracellular matrix and plays an important role in the repair of damaged tissues, contributing to the activation of inflammatory cells and the stimulation of chemokines and cytokines through its interaction with Toll-like receptors [60]. Studies also indicate a potential antifungal effect of hyaluronic acid, which inhibits the growth of *C. albicans* in vitro [61].

### 4.5. The Induction of Genes Involved in the Immune Response of Human Keratinocytes that Encode Compounds with Antimicrobial Activity

Among the functional categories studied, the most important to be evaluated during the fungal-host interaction are those containing the set of genes involved in the human cellular defense. These genes participate not only in the signaling and recruitment of immune system cells, but also in the production of compounds by the host that have a potential antimicrobial effect. These include genes allocated to the MAPK cascade involved in the innate immune response and antimicrobial humoral immune response categories (Table 4).

As an innate cellular defense mechanism, keratinocytes produce peptides with antimicrobial activity, such as cathelicidins, defensins, and ribonucleases [62]. We observed the induction of the *RNASE7* gene (log_2_ fold change: 2.27) that encodes ribonuclease 7. This ribonuclease is known for its marked antimicrobial activity against Gram-positive and -negative bacteria, *C. albicans* [63] and dermatophytes [64], suggesting its use as a new antifungal agent.

Compounds that can be used as new approaches to the treatment of fungal diseases are increasingly being explored because of the growing resistance of pathogenic fungi to conventional antifungal agents [65]. In addition to the *RNASE7* gene, we highlight the induction of the *CSF2* gene (log_2_ fold change: 2.86), which encodes the cytokine granulocyte-macrophage colony-stimulating factor (GM-CSF). Studies indicate the clinical use of this cytokine as an immunological adjuvant for the treatment of fungal diseases. GM-CSF has already been used to treat neutropenic patients undergoing chemotherapy, HIV-infected patients, and bone marrow transplant recipients [66,67]. The effects of this cytokine have been evaluated in species of the genera *Candida* [68] and *Aspergillus* [69], administered alone or in combination with other commercial antifungals.

The induction of the *CSF2* and *RNASE7* genes during co-culture of human keratinocytes with *T. rubrum* may indicate an important cellular defense response of the host when in contact with this fungus since both genes encode compounds with antimicrobial activity. Furthermore, the production of these compounds favors the recruitment of immunocompetent cells to the affected sites that are important for the host’s innate immune mechanisms [63,70,71]

Another gene that was found to be induced in this study and that is also known for its antimicrobial activity is *SLC11A1*. This gene encodes an integral membrane protein [72] that mediates the transport of divalent ions, activating macrophages and exerting other pleiotropic effects on the innate immune system [73]. The available data indicate that this protein protects the host against intracellular pathogens such as *Salmonella* by controlling iron homeostasis inside macrophages, limiting the access of the pathogen to this essential element inside the host, and by concomitantly promoting an increase in the production of antimicrobial effector molecules [72].

Although more elucidated in macrophages, the increased expression of this gene was also observed in keratinocytes of patients with severe burns, suggesting that this gene participates in the innate immune response in the presence of tissue injury [74]. Tissue damage also occurs in dermatophytoses as a result of the secretion of keratinolytic proteases by the fungus. We, therefore, suggest that the induction of this gene during co-culture of keratinocytes with *T. rubrum* may be associated with a defense mechanism of the host, since the *SLC11A1* gene can also exert some signaling effects on the immune system such as macrophage activation, the regulation of interleukin 1-β, and the induction of iNOS, major histocompatibility (MHC) class II molecules, and tumor necrosis factor α (TNFα), among others [75,76]. However, more in-depth studies are necessary to elucidate this possible mechanism of defense

**Figure 4:**
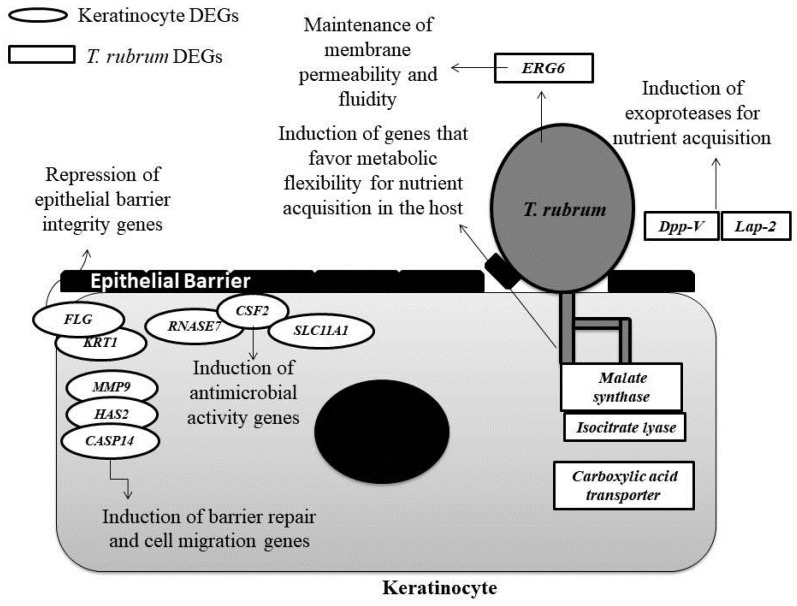
The schematic overview of the *T. rubrum*-keratinocyte interaction. The genes differentially expressed (DEGs) during host-pathogen interaction discussed in this paper are shown.

In summary, within the complex interaction between the fungus and host, we highlight the importance of the modulation of expression of *T. rubrum* genes that contribute to the acquisition and assimilation of nutrients. In this respect, genes responsible for the secretion of keratinolytic proteases (*TERG_12606*; *TERG_08405*) and metabolic adaptation (*TERG_01281*; *TERG_11638*; *TERG_11639*; *TERG_00916*) were found to be induced, as well as the *ERG6* gene that is responsible for maintaining the integrity and permeability of the plasma membrane. In contrast, in the presence of keratinocytes, genes encoding proteins with antimicrobial activity (*RNASE7*; *SLC11A1*; *CSF2*) and genes involved in the maintenance of the skin barrier (*MMP9*; *HAS2*; *CASP14*) are induced, while two genes essential for the stability and integrity of the skin barrier (*FLG*; *KRT1*) are repressed (Figure 4). Considering the limited knowledge, the use of dual RNA-seq allowed for a better understanding of some of the molecular mechanisms involved in the *T. rubrum*-host relationship.

## Figures and Tables

**Table 1:** The major up- and down-regulated genes in HaCat cells after 24 h of co-culture.

ID	Gene Product Name	Log_2_ Fold Change
*SLC9A2*	Sodium/hydrogen exchanger 2	5.01
*ANGPTL4*	Angiopoietin-related protein 4	4.71
*DES*	Desmin	4.53
*C4orf47*	UPF0602 protein C4orf47	4.51
*KISS1R*	KiSS-1 receptor	4.49
*NSA2*	Ribosome biogenesis protein NSA2 homolog	4.35
*HIST1H3C*	Histone cluster 1 H3 family member c	4.04
*SEC11C*	Signal peptidase complex catalytic subunit	3.87
*KPNA7*	Importin subunit alpha-8	3.83
*CASP14*	Caspase 14	3.74
*SLC2A3*	Facilitated glucose transporter member 3	3.73
*ALDOC*	Fructose-bisphosphate aldolase C	3.70
*MT1B*	Metallothionein-1B	3.62
*SERPINE1*	Plasminogen activator inhibitor 1	3.55
*MAF*	Transcription factor Maf	3.54
*CA9*	Carbonic anhydrase 9	3.36
*TGM2*	Transglutaminase 2	3.35
*PADI1*	Protein-arginine deiminase type-1	3.29
*STC1*	Stanniocalcin 1	3.14
*BNIP3*	BCL2 interacting protein 3	3.08
*LSS*	Lanosterol synthase	3.06
*MT1H*	Metallothionein 1H	3.05
*MT1X*	Metallothionein 1X	2.97
*PLA2G2F*	Group IIF secretory phospholipase A2	2.96
*CALB1*	Calbindin 1	2.93
*POTEM*	Putative POTE ankyrin domain family member M	−5.31
*SNORA51*	Small nucleolar RNA. H/ACA box	−4.90
*ANP32A-IT1*	ANP32A intronic transcript 1	−4.64
*UCKL1*	Uridine-cytidine kinase 1 like 1	−4.50
*FNDC3B*	Fibronectin type III domain containing	−4.37
*KRT1*	Keratin 1	−4.02
*MMP12*	Matrix metallopeptidase 12	−3.22
*NSD1*	Nuclear receptor binding SET domain	−3.06
*CYCSP52*	Cytochrome c. somatic pseudogene	−3.02
*EME2*	Essential meiotic structure-specific endonuclease subunit 2	−3.00
*COL12A1*	Collagen type XII alpha 1 chain	−2.88
*SNORD45A*	Small nucleolar RNA. C/D box	−2.82
*FBXL19-AS1*	FBXL19 antisense RNA 1 (head to head)	−2.80
*TRIM26*	Tripartite motif containing 26	−2.76
*IARS*	Isoleucyl-tRNA synthetase	−2.76
*KIF14*	Kinesin family member 14	−2.74
*MEGF8*	Multiple EGF like domains 8	−2.67
*HNRNPL*	Heterogeneous nuclear ribonucleoprotein	−2.66

**Table 2:** The major up- and down-regulated genes in *T. rubrum* after 24 h of co-culture.

ID	Gene Product Name	Log_2_ Fold Change
*TERG_12606*	Dipeptidyl peptidase V (DPPV)	2.16
*TERG_01280*	Hypothetical protein	2.06
*TERG_03102*	Sterol 24-C-methyltransferase- ERG6	2.05
*TERG_08104*	Potassium/sodium efflux P-type ATPase	1.98
*TERG_01281*	Malate synthase	1.72
*TERG_04399*	Phthalate transporter	1.62
*TERG_00215*	MFS peptide transporter	1.47
*TERG_00348*	Galactose-proton symporter	1.47
*TERG_02811*	Hypothetical protein	1.42
*TERG_12645*	Hypothetical protein	1.40
*TERG_07017*	Oxidoreductase	1.35
*TERG_08333*	1-pyrroline-5-carboxylate dehydrogenase	1.34
*TERG_02671*	Hypothetical protein	1.34
*TERG_02023*	Extracellular matrix protein	1.32
*TERG_08405*	Leucine aminopeptidase 2	1.30
*TERG_00916*	Carboxylic acid transporter	1.29
*TERG_11638*	Isocitrate lyase	1.28
*TERG_04952*	ABC transporter	1.26
*TERG_01406*	Hypothetical protein	−2.91
*TERG_07726*	Hypothetical protein	−2.25
*TERG_03174*	MFS siderochrome iron transporter	−1.99
*TERG_06355*	Hypothetical protein	−1.90
*TERG_07035*	Hypothetical protein	−1.85
*TERG_04156*	Hypothetical protein	−1.77
*TERG_05655*	AN1 zinc finger protein	−1.73
*TERG_01622*	Hypothetical protein	−1.63
*TERG_07477*	Hypothetical protein	−1.57
*TERG_06186*	Protein disulfide-isomerase domain-containing protein	−1.57
*TERG_03708*	Hypothetical protein	−1.53
*TERG_03855*	Hypothetical protein	−1.50
*TERG_00499*	Hypothetical protein	−1.45
*TERG_03175*	Hypothetical protein	−1.45
*TERG_04073*	Glutathione synthetase	−1.41
*TERG_12563*	Hypothetical protein	−1.37
*TERG_08139*	NAD dependent epimerase/dehydratase	−1.34
*TERG_06963*	Hsp90-like protein	−1.33
*TERG_01731*	Hypothetical protein	−1.32
*TERG_04006*	Rho guanyl nucleotide exchange factor	−1.32

**Table 3:** Some functional categories and related genes important for the pathogenesis of *T. rubrum.*

ID	Gene Product Name	Log_2_ Fold Change
**Metabolic process**		
*TERG_03102*	Sterol 24-C-methyltransferase	2.05
*TERG_08104*	Sodium transport ATPase	1.98
*TERG_02811*	Hypothetical protein	1.40
*TERG_08333*	Delta 1-pyrroline-5-carboxylate dehydrogenase	1.34
*TERG_11638*	Isocitrate lyase	1.26
*TERG_01270*	AMP-dependent ligase	1.13
*TERG_07691*	Nonspecific lipid-transfer protein	1.13
*TERG_07222*	Carbonic anhydrase	1.05
**Transmembrane transport**		
*TERG_04399*	Phthalate transporter (MFS transporter)	1.62
*TERG_00348*	Galactose-proton symporter (MFS transporter)	1.42
*TERG_00916*	Carboxylic acid transporter (MFS transporter)	1.28
*TERG_04952*	ABC transporter	1.25
*TERG_04356*	Amino acid permease	1.06
**Pathogenesis**		
*TERG_12606*	Dipeptidyl peptidase V	2.16
*TERG_08405*	Leucine Aminopeptidase 2	1.29
**Glyoxylate cycle**		
*TERG_01281*	Malate synthase	1.72
*TERG_11638*	Isocitrate lyase	1.26
*TERG_11639*	Isocitrate lyase	1.13

**Table 4:** Some functional categories and related genes important for human host defense.

ID	Gene Product Name	Log_2_ Fold Change
**Positive regulation of cell migration**		
*TCAF2*	TRPM8 channel-associated factor 2	2.11
*MMP9*	Matrix metalloproteinase-9	2.06
*LAMC2*	Laminin subunit gamma-2	1.97
*HBEGF*	Proheparin-binding EGF-like growth factor	1.84
*HAS2*	Hyaluronan synthase 2	1.46
**MAPK cascade involved in the innate immune response**		
*CSF2*	Granulocyte-macrophage colony-stimulating factor	2.86
*HBEGF*	Proheparin-binding EGF-like growth factor	1.84
*DUSP5*	Dual specificity protein phosphatase 5	1.57
*PSMB3*	Proteasome subunit beta type-3	1.46
*PPP5C*	Serine/threonine-protein phosphatase 5	1.37
*PSMB2*	Proteasome subunit beta type-2	1.28
*UBB*	Polyubiquitin-B	1.24
**Antimicrobial humoral immune response**		
*SERPINE1*	Plasminogen activator inhibitor 1	3.55
*SLC11A1*	Natural resistance-associated macrophage protein 1	2.28
*RNASE7*	Ribonuclease 7	2.27
*RPS19*	40S ribosomal protein S19	1.66
*RPL30*	60S ribosomal protein L30	1.41
**Epidermal cell differentiation**		
*CASP14*	Caspase-14	3.74
*ALDOC*	Fructose-bisphosphate aldolase C	3.70
*AKR1C1*	Aldo-keto reductase family	2.80
*LAMC2*	Laminin subunit gamma-2	1.97
*PGK1*	Phosphoglycerate kinase 1	1.62
**Establishment of the skin barrier**		
*KRT1*	Keratin type II cytoskeletal 1	−4.02
*FLG*	Filaggrin	−1.86

## Supplementary Materials

The following are available online at http://www.mdpi.com/2073-4425/9/7/362/s1, Figure S1: The distribution of differentially expressed genes after 24 h of co-culture. The red points indicate differentially expressed genes. Table S1: The general features of dual RNA-seq sequences against reference genomes. CBS I, CBS II, CBS III: *T. rubrum* libraries; CO I, CO II, CO III: co-culture libraries; H I, H II, H III: human keratinocyte libraries. The libraries were constructed in triplicate, with I, II and III corresponding to the sample number of each condition. PE: paired-end sequence; SR: single read sequence. Table S2: The complete list of genes differentially expressed in keratinocytes after 24 h of co-culture. Table S3: The complete list of genes differentially expressed in *T. rubrum* after 24 h of co-culture. Table S4: The primers used for qPCR analysis.

## References

[1] Bouchara J.P., Mignon B., Chaturvedi V. (2017). Dermatophytes and dermatophytoses: a thematic overview of state of the art, and the directions for future research and developments. Mycopathologia.

[2] Rodwell G.E., Bayles C.L., Towersey L., Aly R. (2008). The prevalence of dermatophyte infection in patients infected with human immunodeficiency virus. Int. J. Dermatol..

[3] Romano C., Massai L., Asta F., Signorini A.M. (2001). Prevalence of dermatophytic skin and nail infections in diabetic patients. Mycoses.

[4] Aly R. (1994). Ecology and epidemiology of dermatophyte infections. J. Am. Acad. Dermatol..

[5] Havlickova B., Czaika V.A., Fredrich M. (2008). Epidemiological trends in skin mycosis worldwide. Mycosis.

[6] Hube B., Hay R., Brasch J., Veraldi S., Schaller M. (2015). Dermatomycoses and inflammation: The adaptive balance between growth, damage, and survival. J. Mycol. Med..

[7] Achterman R.R., White T.C. (2012). A foot in the door for dermatophyte research. PLoS Pathog..

[8] Wolf T., Kämmer P., Brunke S., Linde J. (2018). Two’s company: Studying interspecies relationships with dual RNA-seq. Curr. Opin. Microbiol..

[9] Aprianto R., Slager J., Holsappel S., Veening J.-W. (2016). Time-resolved dual RNA-seq reveals extensive rewiring of lung epithelial and pneumococcal transcriptomes during early infection. Genome Biol..

[10] Wesolowska-Andersen A., Everman J.L., Davidson R., Rios C., Herrin R., Eng C., Janssen W.J., Liu A.H., Oh S.S., Kumar R. (2017). Dual RNA-seq reveals viral infections in asthmatic children without respiratory illness which are associated with changes in the airway transcriptome. Genome Biol..

[11] Tierney L., Linde J., Müller S., Brunke S., Molina J.C., Hube B., Schöck U., Guthke R., Kuchler K. (2012). An interspecies regulatory network inferred from simultaneous RNA-seq of *Candida albicans* invading innate immune cells. Front. Microbiol..

[12] Meyer F.E., Shuey L.S., Naidoo S., Mamni T., Berger D.K., Myburg A.A., Van den Berg N., Naidoo S. (2016). Dual RNA-sequencing of *Eucalyptus nitens* during *Phytophthora cinnamomi* challenge reveals pathogen and host factors influencing compatibility. Front. Plant Sci..

[13] Komoto T.T., Bitencourt T.A., Silva G., Beleboni R.O., Marins M., Fachin A.L. (2015). Gene expression response of *Trichophyton rubrum* during coculture on keratinocytes exposed to antifungal agents. Evid. Based Complement. Altern. Med..

[14] Santiago K., Bomfim G.F., Criado P.R., Almeida S.R. (2014). Monocyte-derived dendritic cells from patients with dermatophytosis restrict the growth of *Trichophyton rubrum* and induce CD4-T cell activation. PLoS ONE.

[15] Edgar R. (2002). Gene Expression Omnibus: NCBI gene expression and hybridization array data repository. Nucleic Acids Res..

[16] Langmead B., Salzberg S.L. (2012). Fast gapped-read alignment with Bowtie 2. Nat. Methods.

[17] Anders S., Huber W. (2010). Differential expression analysis for sequence count data. Genome Biol..

[18] Blake J.A., Harris M.A. (2008). The Gene Ontology (GO) Project: Structured vocabularies for molecular biology and their application to genome and expression analysis. Current Protocols in Bioinformatics.

[19] Gotz S., García-Gómez J.M., Terol J., Williams T.D., Nagaraj S.H., Nueda M.J., Robles M., Talón M., Dopazo J., Conesa A. (2008). High-throughput functional annotation and data mining with the Blast2GO suite. Nucleic Acids Res..

[20] Vêncio R.Z.N., Koide T., Gomes S.L., de Pereira C.A.B. (2006). BayGO: Bayesian analysis of ontology term enrichment in microarray data. BMC Bioinf..

[21] Chen E.Y., Tan C.M., Kou Y., Duan Q., Wang Z., Meirelles G., Clark N.R., Ma’ayan A. (2013). Enrichr: Interactive and collaborative HTML5 gene list enrichment analysis tool. BMC Bioinf..

[22] Kuleshov M.V., Jones M.R., Rouillard A.D., Fernandez N.F., Duan Q., Wang Z., Koplev S., Jenkins S.L., Jagodnik K.M., Lachmann A. (2016). Enrichr: A comprehensive gene set enrichment analysis web server 2016 update. Nucleic Acids Res..

[23] Ma R., Zhang D., Hu P.-C., Li Q., Lin C.-Y. (2015). HOXB7-S3 inhibits the proliferation and invasion of MCF-7 human breast cancer cells. Mol. Med. Rep..

[24] Dai Z., Ma X., Kang H., Gao J., Min W., Guan H., Diao Y., Lu W., Wang X. (2012). Antitumor activity of the selective cyclooxygenase-2 inhibitor, celecoxib, on breast cancer in vitro and in vivo. Cancer Cell Int..

[25] Bitencourt T.A., Komoto T.T., Massaroto B.G., Miranda C.E.S., Beleboni R.O., Marins M., Fachin A.L. (2013). Trans-chalcone and quercetin down-regulate fatty acid synthase gene expression and reduce ergosterol content in the human pathogenic dermatophyte *Trichophyton rubrum*. BMC Complement. Altern. Med..

[26] Jacob T.R., Peres N.T.A., Persinoti G.F., Silva L.G., Mazucato M., Rossi A., Martinez-Rossi N.M. (2012). *Rpb2* is a reliable reference gene for quantitative gene expression analysis in the dermatophyte *Trichophyton rubrum*. Med. Mycol..

[27] Westermann A.J., Barquist L., Vogel J. (2017). Resolving host–pathogen interactions by dual RNA-seq. PLoS Pathog..

[28] Kawahara Y., Oono Y., Kanamori H., Matsumoto T., Itoh T., Minami E. (2012). Simultaneous RNA-seq analysis of a mixed transcriptome of rice and blast fungus interaction. PLoS ONE.

[29] Abadio A.K.R., Kioshima E.S., Teixeira M.M., Martins N.F., Maigret B., Felipe M.S.S. (2011). Comparative genomics allowed the identification of drug targets against human fungal pathogens. BMC Genom..

[30] Baldo A., Monod M., Mathy A., Cambier L., Bagut E.T., Defaweux V., Symoens F., Antoine N., Mignon B. (2012). Mechanisms of skin adherence and invasion by dermatophytes. Mycoses.

[31] Peres N.T.D.A., Maranhão F.C.A., Rossi A., Martinez-Rossi N.M. (2010). Dermatophytes: Host-pathogen interaction and antifungal resistance. An. Bras. Dermatol..

[32] Bitencourt T.A., Macedo C., Franco M.E., Assis A.F., Komoto T.T., Stehling E.G., Beleboni R.O., Malavazi I., Marins M., Fachin A.L. (2016). Transcription profile of *Trichophyton rubrum* conidia grown on keratin reveals the induction of an adhesin-like protein gene with a tandem repeat pattern. BMC Genom..

[33] Monod M., Léchenne B., Jousson O., Grand D., Zaugg C., Stöcklin R., Grouzmann E. (2005). Aminopeptidases and dipeptidyl-peptidases secreted by the dermatophyte *Trichophyton rubrum*. Microbiology.

[34] Leng W., Liu T., Wang J., Li R., Jin Q. (2009). Expression dynamics of secreted protease genes in *Trichophyton rubrum* induced by key host’s proteinaceous components. Med. Mycol..

[35] Azam S.S., Abro A., Raza S., Saroosh A. (2014). Structure and dynamics studies of sterol 24-C-methyltransferase with mechanism based inactivators for the disruption of ergosterol biosynthesis. Mol. Biol. Rep..

[36] Iwaki T., Iefuji H., Hiraga Y., Hosomi A., Morita T., Giga-Hama Y., Takegawa K. (2008). Multiple functions of ergosterol in the fission yeast *Schizosaccharomyces pombe*. Microbiology.

[37] Osborne C.S., Leitner I., Favre B., Neil S., Osborne C.S., Leitner I., Favre B., Ryder N.S. (2005). Amino acid substitution in *Trichophyton rubrum* squalene epoxidase associated with resistance to terbinafine.

[38] Gaber R.F., Copple D.M., Kennedy B.K., Vidal M., Bard M. (1989). The yeast gene *ERG6* is required for normal membrane function but is not essential for biosynthesis of the cell-cycle-sparking sterol. Mol. Cell. Biol..

[39] Mayer F.L., Wilson D., Hube B. (2013). *Candida albicans* pathogenicity mechanisms. Virulence.

[40] Cheah H.L., Lim V., Sandai D. (2014). Inhibitors of the glyoxylate cycle enzyme ICL1 in *Candida albicans* for potential use as antifungal agents. PLoS ONE.

[41] Fleck C.B., Schöbel F., Brock M. (2011). Nutrient acquisition by pathogenic fungi: Nutrient availability, pathway regulation, and differences in substrate utilization. Int. J. Med. Microbiol..

[42] Cantelli B.A.M., Bitencourt T.A., Komoto T.T., Beleboni R.O., Marins M., Fachin A.L. (2017). Caffeic acid and licochalcone A interfere with the glyoxylate cycle of *Trichophyton rubrum*. Biomed. Pharmacother..

[43] Vieira N., Casal M., Johansson B., MacCallum D.M., Brown A.J.P., Paiva S. (2010). Functional specialization and differential regulation of short-chain carboxylic acid transporters in the pathogen *Candida albicans*. Mol. Microbiol..

[44] Lee W.J., Kim J.Y., Song C.H., Jung H.D., Lee S.H., Lee S.J., Kim D.W. (2011). Disruption of barrier function in dermatophytosis and pityriasis versicolor. J. Dermatol..

[45] Brown S.J., Irvine A.D. (2008). Atopic eczema and the filaggrin story. Semin. Cutan. Med. Surg..

[46] McGrath J.A. (2008). Filaggrin and the great epidermal barrier grief. Australas. J. Dermatol..

[47] Jensen J.-M., Pfeiffer S., Akaki T., Schröder J.-M., Kleine M., Neumann C., Proksch E., Brasch J. (2007). Barrier function, epidermal differentiation, and human β-defensin 2 expression in Tinea Corporis. J. Investig. Dermatol..

[48] Roth W., Kumar V., Beer H.-D., Richter M., Wohlenberg C., Reuter U., Thiering S., Staratschek-Jox A., Hofmann A., Kreusch F. (2012). Keratin 1 maintains skin integrity and participates in an inflammatory network in skin through interleukin-18. J. Cell Sci..

[49] Parks W., Wilson C., López-Boado Y. (2004). Matrix metalloproteinases as modulators of inflammation and innate immunity. Nat. Rev. Immunol..

[50] Purwar R., Kraus M., Werfel T., Wittmann M. (2008). Modulation of keratinocyte-derived MMP-9 by IL-13: A possible role for the pathogenesis of epidermal inflammation. J. Investig. Dermatol..

[51] Hvid M., Johansen C., Deleuran B., Kemp K., Deleuran M., Vestergaard C. (2011). Regulation of caspase 14 expression in keratinocytes by inflammatory cytokines—A possible link between reduced skin barrier function and inflammation?. Exp. Dermatol..

[52] Gkegkes I.D., Aroni K., Agrogiannis G., Patsouris E.S., Konstantinidou A.E. (2013). Expression of caspase-14 and keratin-19 in the human epidermis and appendages during fetal skin development. Arch. Dermatol. Res..

[53] Lippens S., Kockx M., Knaapen M., Mortier L., Polakowska R., Verheyen A., Garmyn M., Zwijsen A., Formstecher P., Huylebroeck D. (2000). Epidermal differentiation does not involve the pro-apoptotic executioner caspases, but is associated with caspase-14 induction and processing. Cell Death Differ..

[54] Denecker G., Hoste E., Gilbert B., Hochepied T., Ovaere P., Lippens S., Van den Broecke C., Van Damme P., D’Herde K., Hachem J.-P. (2007). Caspase-14 protects against epidermal UVB photodamage and water loss. Nat. Cell Biol..

[55] Ratzinger G., Stoitzner P., Ebner S., Lutz M.B., Layton G.T., Rainer C., Senior R.M., Shipley J.M., Fritsch P., Schuler G. (2002). Matrix metalloproteinases 9 and 2 are necessary for the migration of langerhans cells and dermal dendritic cells from human and murine skin. J. Immunol..

[56] Visse R., Nagase H. (2003). Matrix metalloproteinases and tissue inhibitors of metalloproteinases: Structure, function, and biochemistry. Circ. Res..

[57] Lee S., Zheng M., Kim B., Rouse B.T. (2002). Role of matrix metalloproteinase-9 in angiogenesis caused by ocular infection with herpes simplex virus. J. Clin. Investig..

[58] Campos T.M., Passos S.T., Novais F.O., Beiting D.P., Costa R.S., Queiroz A., Mosser D., Scott P., Carvalho E.M., Carvalho L.P. (2014). Matrix metalloproteinase 9 production by monocytes is enhanced by TNF and participates in the pathology of human Cutaneous Leishmaniasis. PLoS Negl. Trop. Dis..

[59] Murphy G., Nagase H. (2009). Progress in matrix metalloproteinase research. Mol. Aspects Med..

[60] Jiang D., Liang J., Noble P.W. (2007). Hyaluronan in tissue injury and repair. Annu. Rev. Cell Dev. Biol..

[61] Sakai A., Akifusa S., Itano N., Kimata K., Kawamura T., Koseki T., Takehara T., Nishihara T. (2007). Potential role of high molecular weight hyaluronan in the anti-*Candida* activity of human oral epithelial cells. Med. Mycol..

[62] Becknell B., Spencer J.D. (2016). A Review of ribonuclease 7’s structure, regulation, and contributions to host defense. Int. J. Mol. Sci..

[63] Harder J., Schröder J.M. (2002). RNase 7, a novel innate immune defense antimicrobial protein of healthy human skin. J. Biol. Chem..

[64] Fritz P., Beck-Jendroschek V., Brasch J. (2012). Inhibition of dermatophytes by the antimicrobial peptides human β-defensin-2, ribonuclease 7 and psoriasin. Med. Mycol..

[65] Mehra T., Köberle M., Braunsdorf C., Mailänder-Sanchez D., Borelli C., Schaller M. (2012). Alternative approaches to antifungal therapies. Exp. Dermatol..

[66] Hubel K., Dale D.C., Liles W.C. (2002). Therapeutic use of cytokines to modulate phagocyte function for the treatment of infectious diseases: Current status of granulocyte colony-stimulating factor, granulocyte-macrophage colony-stimulating factor, macrophage colony-stimulating factor, and interferon-gamma. J. Infect. Dis..

[67] Shi Y., Liu C.H., Roberts A.I., Das J., Xu G., Ren G., Zhang Y., Zhang L., Yuan Z.R., Tan H.S.W. (2006). Granulocyte-macrophage colony-stimulating factor (GM-CSF) and T-cell responses: What we do and don’t know. Cell Res..

[68] Liehl E., Hildebrandt J., Lam C., Mayer P. (1994). Prediction of the role of granulocyte-macrophage colony-stimulating factor in animals and man from in vitro results. Eur. J. Clin. Microbiol. Infect. Dis..

[69] Bodey G.P., Anaissie E., Gutterman J., Vadhan-Raj S. (1994). Role of granulocyte-macrophage colony-stimulating factor as adjuvant treatment in neutropenic patients with bacterial and fungal infection. Eur. J. Clin. Microbiol. Infect. Dis..

[70] Hamilton J.A. (2002). GM-CSF in inflammation and autoimmunity. Trends Immunol..

[71] Rademacher F., Simanski M., Harder J. (2016). RNase 7 in cutaneous defense. Int. J. Mol. Sci..

[72] Nairz M., Fritsche G., Crouch M.L.V., Barton H.C., Fang F.C., Weiss G. (2009). Slc11a1 limits intracellular growth of *Salmonella enterica* sv. Typhimurium by promoting macrophage immune effector functions and impairing bacterial iron acquisition. Cell. Microbiol..

[73] Stober C.B., Brode S., White J.K., Popoff J.-F., Blackwell J.M. (2007). Slc11a1, formerly Nramp1, is expressed in dendritic cells and influences major histocompatibility complex class II expression and antigen-presenting cell function. Infect. Immun..

[74] Noronha S.A., Noronha S.M., Lanziani L.E., Ferreira L.M., Gragnani A. (2014). Innate and adaptive immunity gene expression of human keratinocytes cultured of severe burn injury. Acta Cir. Bras..

[75] Blackwell J.M., Searle S., Goswami T., Miller E.N. (2000). Understanding the multiple functions of Nrampl. Microbes Infect..

[76] Blackwell J.M., Goswami T., Evans C.A.W., Sibthorpe D., Papo N., White J.K., Searle S., Miller E.N., Peacock C.S., Mohammed H. (2001). SLC11A1 (formerly NRAMP1) and disease resistance. Cell. Microbiol..

