# Supplementary material for: Dual RNA-Seq Analysis of Trichophyton rubrum and HaCat Keratinocyte Co-Culture Highlights Important Genes for Fungal-Host Interaction
Source: Genes (Basel). 2018 Jul 19;9(7):362. doi: 10.3390/genes9070362 (PMC6070946; doi:10.3390/genes9070362)
Supplement: Supplementary file 1 [file genes-09-00362-s001.zip › Supplementary Table S4_.pdf]

Supplementary Table S4. Primers used for qPCR analysis.

ID	Gene product name	Primer sequence (5'- 3')	Fragment	Reference
TERG_12606	Dipeptidyl peptidase V	F:CGGTTTCGATAAGAACAAGGTC<br>R:ACAACGTATCCCTGGTCAG	126 bp	This paper
TERG_03102	Sterol 24-C-methyltransferase	F:CTCTGGCAAGACACGAACAC<br>R:CCTTGCAGCCGGTGAAGG	126 bp	Bitencourt et al., 2013
TERG_01281	Malate synthase	F: CCTAACCAACTCTTTGTCCGAC<br>R: GAAGCCATCCCTCCATGTAAG	142 bp	Cantelli et al., 2017
TERG_08405	Leucine aminopeptidase 2	F:GCTGCACAAGGTCAAGTTG<br>R:ACTTCTCGAACAGGTACTCG	158 bp	This paper
TERG_00916	Carboxylic acid transporter	F:CGCATTGGGTATCATGATCAC<br>R:GCTCTTGTTCACCGTCATG	109 bp	This paper
TERG_11638	Isocitrate lyase	F:ACAACCTCTCGCCTTCATTC<br>R:GGTCAGATATCAGGGCAGTTG	144 bp	Komoto et al., 2015
TERG_07904	Tubulin beta chain	F:AACATGATGGCTGCCACTGA<br>R:AAGATGGCAGAGCAGGTAAGGT	253 bp	Jacob et al., 2012
TERG08193	18S	F:CGCTGGCTTCTTAGAGGGACTAT<br>R:TGCCTCAAACTTCCATCGACTT	51 bp	Bitencourt et al., 2013
CASP14	Caspase-14	F:CATCAAAGACAGCCCACAAAC<br>R:TGTCCTTTCCTCTTCGTGAAC	150 bp	This paper
CSF2	Granulocyte-macrophage colony-stimulating factor	F:TCTGCTTGTCATCCCCTTTG<br>R:ATGACCATCCTGAGTTTCTAGC	122 bp	Kim et al., 2016
RNASE7	Ribonuclease 7	F:GGAGTCACAGCACGAAGACCA<br>R:CATGGCTGAGTTGCATGCTTGA	235 bp	Köten et al., 2009
SLC11A1	Natural resistance-associated macrophage protein 1	F:CATCTCCGTCTCCCAAAGTG<br>R:TGGATGCTTTAGAGACTTCGTG	144 bp	This paper

MMP9	Matrix metalloproteinase 9	F:CAGTTTCCATTCATCTTCCAAGG<br>R:CATCACCGTCGAGTCAGC	150 bp	Markiewicz et al., 2006
HAS2	Hyaluronan synthase 2	F:TCCGAGAATGGCTGTACAATG<br>R:AAGAGCTGGATTACTGTGGC	118 bp	This paper
KRT1	Keratin type II cytoskeletal 1	F:CCTTACAGCACTCTACCAAGC<br>R:TCTGGGCTATATCCTCGTACTG	149 bp	This paper
FLG	Filaggrin	F:AGCAATCGGTAAATAGGTCTGG<br>R:CCTCATTTCGTGTTTGTCTGC	126 bp	This paper
GAPDH	Glyceraldehyde-3-phosphate dehydrogenase	F:AATCCCATCACCATCTTCCAG<br>R:GAGCCCCAGCCTTCTCCAT	118 bp	Ma et al., 2015
ACTB	Beta-actin	F:GTTGCGTTACACCCTTTCTTG<br>R:TGCTGTCACCTTCACCGTTC	154 bp	Dai et al., 2012
