# Supplementary material for: Dual RNA-Seq Analysis of Trichophyton rubrum and HaCat Keratinocyte Co-Culture Highlights Important Genes for Fungal-Host Interaction
Source: Genes (Basel). 2018 Jul 19;9(7):362. doi: 10.3390/genes9070362 (PMC6070946; doi:10.3390/genes9070362)
Supplement: Supplementary file 1 [file genes-09-00362-s001.zip › Supplementary Material.pdf]

Supplementary Material

Dual RNA-seq analysis of *Trichophyton rubrum* and HaCat keratinocytes co-culture highlights important genes for fungal-host interactions

RUNNING TITLE: Dual RNA-seq of *T. rubrum*-keratinocyte interaction

AUTHORS: Monise Fazolin Petrucelli[1], Kamila Peronni[4], Pablo Rodrigo Sanches[6], Tatiana Takahasi Komoto[1], Josie Budag Matsuda[1], Rene Oliveira Beleboni[1,2], Nilce Maria Martinez-Rossi[6], Wilson Araújo da Silva Junior[3,4,5], Mozart Marins[1,2], Ana Lúcia Fachin [1,2]*

[1] Biotechnology Unit, University of Ribeirão Preto-UNAERP, Brazil.
[2] Medicine School, University of Ribeirão Preto- UNAERP, Brazil

[3] Department of Genetics, Ribeirão Preto Medical School, and Center for Integrative System Biology - CISBi-NAP/USP, University of São Paulo, Ribeirão Preto, São Paulo, Brazil.

[4] National Institute of Science and Technology in Stem Cell and Cell Therapy and Center for Cell-Based Therapy, Ribeirão Preto, São Paulo, Brazil.

[5] Center for Medical Genomics at Clinical Hospital of the Medical School of Ribeirão Preto, University of São Paulo, Ribeirão Preto, SP, Brazil.

[6] Department of Genetics, Ribeirão Preto Medical School, University of São Paulo, Ribeirão Preto, Brazil

***Corresponding author**: Ana Lúcia Fachin – Biotechnology Unit, University of Ribeirão Preto. Av: Costábile Romano 2201, 14096-900, Ribeirão Preto, SP, Brazil FAX + 55 -16-36037030. email: afachin@unaerp.br

1 Supplementary Figures and Tables

Supplementary Figure S1: Distribution of differentially expressed genes after 24 h of co-culture. The red points indicate differentially expressed genes.

Supplementary Table S1: General features of dual RNA-seq sequences against reference genomes. CBS I, CBS II, CBS III: *T. rubrum* libraries; CO I, CO II, CO III: co-culture libraries; H I, H II, H III: human keratinocyte libraries. The libraries were constructed in triplicate, with I, II and III corresponding to the sample number of each condition. PE: paired-end sequence; SR: single read sequence.

Supplementary Table S2: Complete list of genes differentially expressed in keratinocytes after 24 h of co-culture.

Supplementary Table S3: Complete list of genes differentially expressed in *T. rubrum* after 24 h of co-culture.

Supplementary Table S4: Primers used for qPCR analysis.
