# Supplementary material for: Dual RNA-Seq Analysis of Trichophyton rubrum and HaCat Keratinocyte Co-Culture Highlights Important Genes for Fungal-Host Interaction
Source: Genes (Basel). 2018 Jul 19;9(7):362. doi: 10.3390/genes9070362 (PMC6070946; doi:10.3390/genes9070362)
Supplement: Supplementary file 1 [file genes-09-00362-s001.zip › Supplementary Table S1.pdf]

Supplementary Table S1. General features of dual RNA-seq sequences against reference genomes.

			T. rubrum **reference genome (CBS 118892)**		**Human reference genome (HG19)**	
**Sample**	**Raw reads**	**High-quality reads**	**Mapped reads**	**Mapped reads (%)**	**Mapped reads**	**Mapped reads (%)**
CBS I (PE)	60,462,551	59,066,317	47,766,930	80.87%	-	-
CBS II (SR)	37,501,479	36,676,227	31,692,446	86.41%	-	-
CBS III (SR)	27,677,792	27,063,599	24,635,560	91.03%	-	-
CO I (PE)	50,412,147	48,758,632	351,062	0.72%	41,752,016	85.63%
CO II (SR)	27,926,550	27,331,638	2,571,234	9.41%	22,298,067	81.58%
CO III (SR)	25,574,109	25,063,089	1,388,085	5.54%	21,400,355	85.39%
H I (SR)	35,308,490	34,530,495	-	-	30,625,029	88.69%
H II (PE)	61,220,210	58,106,283	-	-	48,611,716	83.66%
H III (PE)	45,385,154	42,869,051	-	-	35,992,855	83.96%

CBS I, CBS II, CBS III: *T. rubrum* libraries; CO I, CO II, CO III: co-culture libraries; H I, H II, H III: human keratinocyte libraries. The libraries were constructed in triplicate, with I, II and III corresponding to the sample number of each condition. PE: paired-end sequence; SR: single read sequence.
