# Supplementary material for: Dual RNA-Seq Analysis of Trichophyton rubrum and HaCat Keratinocyte Co-Culture Highlights Important Genes for Fungal-Host Interaction
Source: Genes (Basel). 2018 Jul 19;9(7):362. doi: 10.3390/genes9070362 (PMC6070946; doi:10.3390/genes9070362)
Supplement: Supplementary file 1 [file genes-09-00362-s001.zip › Supplementary Table S2_.pdf]

Supplementary Table S2. Complete list of genes differentially expressed in keratinocytes after 24 h of co-culture.

ID	Gene product name	Log_2 fold change
SLC9A2	Solute carrier family 9 member A2	5.0
ANGPTL4	Angiopoietin like 4	4.7
DES	Desmin	4.5
C4orf47	Chromosome 4 open reading frame 47	4.5
KISS1R	KISS1 receptor	4.5
NSA2	NSA2, ribosome biogenesis homolog	4.4
HIST1H3C	Histone cluster 1 H3 family member c	4.0
SEC11C	SEC11 homolog C, signal peptidase complex subunit	3.9
KPNA7	Karyopherin subunit alpha 7	3.8
CASP14	Caspase 14	3.7
SLC2A3	Solute carrier family 2 member 3	3.7
ALDOC	Aldolase, fructose-bisphosphate C	3.7
MT1B	Metallothionein 1B	3.6
SERPINE1	Serpin family E member 1	3.6
MAF	MAF bzip transcription factor	3.5
CA9	Carbonic anhydrase 9	3.4
TGM2	Transglutaminase 2	3.4
PADI1	Peptidyl arginine deiminase 1	3.3
STC1	Stanniocalcin 1	3.1
BNIP3	BCL2 interacting protein 3	3.1
LSS	Lanosterol synthase	3.1
MT1H	Metallothionein 1H	3.1
MT1JP	Metallothionein 1J, pseudogene	3.0
MYH16	Myosin heavy chain 16 pseudogene	3.0
MT1X	Metallothionein 1X	3.0
PLA2G2F	Phospholipase A2 group IIF	3.0

CALB1	Calbindin 1	2.9
KRT42P	Keratin 42 pseudogene	2.9
ENO2	Enolase 2	2.9
ZFP90	ZFP90 zinc finger protein	2.9
VEZT	Vezatin, adherens junctions transmembrane protein	2.9
CSF2	Colony stimulating factor 2	2.9
MT1L	Metallothionein 1L, pseudogene	2.8
CCNG1	Cyclin G1	2.8
AKR1C1	Aldo-keto reductase family 1 member C1	2.8
RGCC	Regulator of cell cycle	2.8
WFDC3	WAP four-disulfide core domain 3	2.8
KRT17P5	Keratin 17 pseudogene 5	2.8
C20orf195(FNDC11)	Fibronectin type III domain containing 11	2.7
PFKFB4	6-phosphofructo-2-kinase/fructose-2,6-biphosphatase 4	2.7
WISP2	WNT1 inducible signaling pathway protein 2	2.7
SNRPD2P2	Small nuclear ribonucleoprotein D2 pseudogene 2	2.6
FZD10	Frizzled class receptor 10	2.6
KCTD11	Potassium channel tetramerization domain containing 11	2.6
CLIC3	Chloride intracellular channel 3	2.6
EFNA1	Ephrin A1	2.5
MTRNR2L8	MT-RNR2-like 8	2.5
WIPI1	WD repeat domain, phosphoinositide interacting	2.5
RPL17	Ribosomal protein L17	2.4
ITGA5	Integrin subunit alpha 5	2.4
RPLP1	Ribosomal protein lateral stalk subunit P1	2.4
KIAA1755	Kiaa1755	2.4
AKR1C4	Aldo-keto reductase family 1 member C4	2.4
LOXL2	Lysyl oxidase like 2	2.3
ABCA3	ATP binding cassette subfamily A member 3	2.3

MT1A	Metallothionein 1[a]	2.3
RPS27	Ribosomal protein S27	2.3
SLC11A1	Natural resistance-associated macrophage protein 1	2.3
RAB11A	RAB11A, member RAS oncogene family	2.3
MTRNR2L5	MT-RNR2-like 5	2.3
RNASE7	Ribonuclease 7	2.3
DDIT4	DNA damage inducible transcript 4	2.2
RASSF7	Ras association domain family member 7	2.2
TBCA	Tubulin folding cofactor A	2.2
TXN	Thioredoxin	2.2
INSIG1	Insulin induced gene 1	2.2
RPS29	Ribosomal protein S29	2.2
CRIP1	Cysteine rich protein 1	2.2
MT2A	Metallothionein 2A	2.2
SEPT8	Septin 8	2.2
GJB4	Gap junction protein beta 4	2.2
RPSAP52	Ribosomal protein SA pseudogene	2.1
APOBEC3G	Apolipoprotein B mRNA editing enzyme catalytic subunit 3G	2.1
EGLN3	Egl-9 family hypoxia inducible factor 3	2.1
AGBL5	ATP/GTP binding protein like 5	2.1
MTRNR2L6	MT-RNR2-like 6	2.1
TCAF2	TRPM8 channel associated factor 2	2.1
MRPS18A	Mitochondrial ribosomal protein	2.1
FTMT	Ferritin mitochondrial	2.1
EMP3	Epithelial membrane protein 3	2.1
S100P	S100 calcium binding protein P	2.1
KRT84	Keratin 84	2.1
MMP9	Matrix metallopeptidase 9	2.1
ALG5	Dolichyl-phosphate beta-glucosyltransferase	2.1

PAG1	Phosphoprotein membrane anchor with glycosphingolipid microdomains 1	2.1
TINF2	TERF1 interacting nuclear factor 2	2.0
RPS21	Ribosomal protein S21	2.0
NENF	Neudesin neurotrophic factor	2.0
BNIP3L	BCL2 interacting protein 3 like	2.0
SMS	Spermine synthase	2.0
KRT16P3	Keratin 16 pseudogene 3	2.0
CDA	Cytidine deaminase	2.0
PPIAL4G	Peptidylprolyl isomerase A like 4G	2.0
SHROOM4	Shroom family member 4	2.0
MTRNR2L4	MT-RNR2-like 4	2.0
LAMC2	Laminin subunit gamma 2	2.0
OSGIN1	Oxidative stress induced growth inhibitor	2.0
SENP6	SUMO1/sentrin specific peptidase 6	2.0
ATP5I	ATP synthase, H+ transporting, mitochondrial Fo complex subunit E	2.0
IGFL1	IGF like family member 1	1.9
AMY2B	Amylase, alpha 2B (pancreatic)	1.9
RPS18	Ribosomal protein S18	1.9
HK2	Hexokinase 2	1.9
ANKRD37	Ankyrin repeat domain 37	1.9
SUMO2	Small ubiquitin-like modifier 2	1.9
FAM83A	Family with sequence similarity 83 member A	1.9
JUN	Jun proto-oncogene, AP-1 transcription factor subunit	1.9
BHLHE40	Basic helix-loop-helix family member 40	1.9
ADAM19	ADAM metallopeptidase domain 19	1.9
TMC8	Transmembrane channel like 8	1.8
MSC	Musculin	1.8
RPL12	Ribosomal protein L12	1.8
HBEGF	Heparin binding EGF like growth factor	1.8

ARNT2	Aryl hydrocarbon receptor nuclear translocator 2	1.8
PPP2R5B	Protein phosphatase 2 regulatory subunit b'beta	1.8
RNF181	Ring finger protein 181	1.8
BRMS1	Breast cancer metastasis suppressor 1	1.8
CDK7	Cyclin dependent kinase 7	1.8
FTH1P3	Ferritin heavy chain 1 pseudogene 3	1.8
EIF3L	Eukaryotic translation initiation factor 3 subunit L	1.8
LBH	Limb bud and heart development	1.8
FGFBP1	Fibroblast growth factor binding protein 1	1.8
CYP1A1	Cytochrome P450 family 1 subfamily A member 1	1.8
ADM	Adrenomedullin	1.8
RPS12	Ribosomal protein S12	1.7
NHP2	NHP2 ribonucleoprotein	1.7
RPS28	Ribosomal protein S28	1.7
RPL10	Ribosomal protein L10	1.7
MVD	Mevalonate diphosphate decarboxylase	1.7
RGS20	Regulator of G-protein signaling 20	1.7
EXOSC10	Exosome component 10	1.7
RPL37A	Ribosomal protein L37A	1.7
SYNGR3	Synaptogyrin 3	1.7
RPL39	Ribosomal protein L39	1.7
MTRNR2L9	MT-RNR2-like 9	1.7
DAPK2	Death associated protein kinase 2	1.7
RPL23	Ribosomal protein L23	1.7
AKR1B10	Aldo-keto reductase family 1 member B10	1.7
CHMP5	Charged multivesicular body protein 5	1.7
RPS19	Ribosomal protein S19	1.7
FAM83A-AS1	FAM83A antisense RNA 1	1.7
RPS16	Ribosomal protein S16	1.6

FAM162A	Family with sequence similarity 162 member A	1.6
ATP5H	ATP synthase, H+ transporting, mitochondrial Fo complex subunit D	1.6
RPS7	Ribosomal protein S7	1.6
CT62	Cancer/testis antigen 62	1.6
HMOX1	Heme oxygenase 1	1.6
PNRC1	Proline rich nuclear receptor coactivator 1	1.6
GOLGA7B	Golgin A7 family member B	1.6
LOC344887	NmrA like redox sensor 2, pseudogene	1.6
WDR54	WD repeat domain 54	1.6
PGK1	Phosphoglycerate kinase 1	1.6
INSIG2	Insulin induced gene 2	1.6
APCDD1L-AS1	APCDD1L antisense RNA 1 (head to head)	1.6
TMBIM1	Transmembrane BAX inhibitor motif containing 1	1.6
RAB2A	RAB2A, member RAS oncogene family	1.6
TMEM45A	Transmembrane protein 45[a]	1.6
FAM214B	Family with sequence similarity 214 member B	1.6
CASC8	Cancer susceptibility 8 (non-protein coding)	1.6
RPL24	Ribosomal protein L24	1.6
MMD	Monocyte to macrophage differentiation associated	1.6
DUSP5	Dual specificity phosphatase 5	1.6
LGALS1	Galectin 1	1.6
MMP28	Matrix metallopeptidase 28	1.6
SNHG5	Small nucleolar RNA host gene 5	1.6
RPL19	Ribosomal protein L19	1.6
PRSS22	Protease, serine 22	1.6
JAZF1	JAZF zinc finger 1	1.5
EFNA3	Ephrin A3	1.5
RPS25	Ribosomal protein S25	1.5
IER3	Immediate early response 3	1.5

BIK	BCL2 interacting killer	1.5
ERO1A	Endoplasmic reticulum oxidoreductase 1 alpha	1.5
TP53INP2	Tumor protein p53 inducible nuclear protein 2	1.5
RPL35A	Ribosomal protein l35a	1.5
RRAS	Related RAS viral (r-ras) oncogene homolog	1.5
GBAS	Nipsnap homolog 2	1.5
KIF3C	Kinesin family member 3C	1.5
IDI1	Isopentenyl-diphosphate delta isomerase 1	1.5
RPL27A	Ribosomal protein l27 A	1.5
TMEM120A	Transmembrane protein 120A	1.5
PPIH	Peptidylprolyl isomerase H	1.5
VKORC1	Vitamin K epoxide reductase complex subunit 1	1.5
RPS11	Ribosomal protein S11	1.5
RTN4R	Reticulon 4 receptor	1.5
RPLP2	Ribosomal protein lateral stalk subunit P2	1.5
WNT7A	Wnt family member 7A	1.5
CYP27B1	Cytochrome P450 family 27 subfamily B member 1	1.5
RPL18A	Ribosomal protein l18a	1.5
PSMB3	Proteasome subunit beta 3	1.5
RPL5	Ribosomal protein L5	1.5
HAS2	Hyaluronan synthase 2	1.5
ACTG1P17	Actin gamma 1 pseudogene 17	1.5
FNTA	Farnesyltransferase, CAAX box, alpha	1.4
MUC13	Mucin 13, cell surface associated	1.4
IGIP	Iga inducing protein	1.4
FOS	Fos proto-oncogene, AP-1 transcription factor subunit	1.4
BTG1	BTG anti-proliferation factor 1	1.4
SNHG6	Small nucleolar RNA host gene 6	1.4
GAS5	Growth arrest specific 5 (non-protein coding)	1.4

RPL30	Ribosomal protein L30	1.4
MICALCL	MICAL C-terminal like	1.4
PPP2R5C	Protein phosphatase 2 regulatory subunit b'gamma	1.4
RPS8	Ribosomal protein S8	1.4
RPL35	Ribosomal protein L35	1.4
RPL13AP20	Ribosomal protein L13a pseudogene 20	1.4
PPP5C	Protein phosphatase 5 catalytic subunit	1.4
HMGN2P46	High mobility group nucleosomal binding domain 2 pseudogene 46	1.4
RPL7A	Ribosomal protein l7A	1.4
RPL26	Ribosomal protein L26	1.3
RPS4X	Ribosomal protein S4, X-linked	1.3
RPS13	Ribosomal protein S13	1.3
RPL27	Ribosomal protein L27	1.3
RPS6	Ribosomal protein S6	1.3
RPL37	Ribosomal protein L37	1.3
PSMB2	Proteasome subunit beta 2	1.3
COX7B	Cytochrome c oxidase subunit 7B	1.3
RPL3	Ribosomal protein L3	1.3
EIF3E	Eukaryotic translation initiation factor 3 subunit E	1.2
UBB	Ubiquitin B	1.2
RPS3A	Ribosomal protein S3A	1.2
RPL7	Ribosomal protein L7	1.2
POTEI	POTE ankyrin domain family member I	1.2
POTEM	POTE ankyrin domain family member	-5.3
SNORA51	Small nucleolar RNA, H/ACA box	-4.9
ANP32A-IT1	ANP32A intronic transcript 1	-4.6
UCKL1	Uridine-cytidine kinase 1 like 1	-4.5
FNDC3B	Fibronectin type III domain containing	-4.4
LOC729739	Peptidylprolyl isomerase A (cyclophilin A) pseudogene	-4.3

RPL23AP64	Ribosomal protein L23a pseudogene	-4.2
KRT1	Keratin 1	-4.0
FDPSP2	Farnesyl diphosphate synthase pseudogene 2	-3.2
MMP12	Matrix metallopeptidase 12	-3.2
NSD1	Nuclear receptor binding SET domain	-3.1
CYCSP52	Cytochrome c, somatic pseudogene	-3.0
EME2	Essential meiotic structure-specific endonuclease subunit 2	-3.0
COL12A1	Collagen type XII alpha 1 chain	-2.9
RPL23AP32	Ribosomal protein L23a pseudogene	-2.9
HSP90AB4P	Heat shock protein 90 alpha family class B member 4, pseudogene	-2.8
SNORD45A	Small nucleolar RNA, C/D box	-2.8
FBXL19-AS1	FBXL19 antisense RNA 1 (head to head)	-2.8
TRIM26	Tripartite motif containing 26	-2.8
IARS	Isoleucyl-tRNA synthetase	-2.8
KIF14	Kinesin family member 14	-2.7
IFITM4P	Interferon induced transmembrane protein 4 pseudogene	-2.7
MEGF8	Multiple EGF like domains 8	-2.7
HNRNPL	Heterogeneous nuclear ribonucleoprotein	-2.7
SPTBN5	Spectrin beta, non-erythrocytic 5	-2.7
SOX2	SRY-box 2	-2.6
KIAA0586	Kiaa0586	-2.6
RBP5	Retinol binding protein 5	-2.6
AGRN	Agrin	-2.6
CAD	Carbamoyl-phosphate synthetase 2, aspartate transcarbamylase, and dihydroorotase	-2.5
C9orf172	Chromosome 9 open reading frame	-2.5
ANKRD30BL	Ankyrin repeat domain 30B like	-2.4
ITPRIPL2	Inositol 1,4,5-trisphosphate receptor interacting protein like 2	-2.4
SMC4	Structural maintenance of chromosomes 4	-2.4
MKI67	Marker of proliferation Ki-67	-2.4

EIF5A	Eukaryotic translation initiation factor 5A	-2.4
LMNB1	Lamin B1	-2.3
DNAH11	Dynein axonemal heavy chain 11	-2.3
EIF5	Eukaryotic translation initiation factor 5	-2.3
MSH2	Muts homolog 2	-2.2
SREK1	Splicing regulatory glutamic acid and lysine rich protein 1	-2.2
IGSF9B	Immunoglobulin superfamily member 9B	-2.2
UGT8	UDP glycosyltransferase 8	-2.2
PWAR5	Prader Willi/Angelman region RNA 5	-2.2
C1RL-AS1	C1RL antisense RNA 1	-2.1
UCKL1-AS1	UCKL1 antisense RNA 1	-2.1
ACTG1P4	Actin gamma 1 pseudogene 4	-2.1
TNPO2	Transportin 2	-2.1
PUS1	Pseudouridylate synthase 1	-2.1
LOC100130331	POTE ankyrin domain family, member F	-2.1
RACGAP1P	Rac gtpase activating protein 1	-2.1
NAT10	N-acetyltransferase 10	-2.1
ECT2	Epithelial cell transforming 2	-2.0
SLC25A2	Solute carrier family 25 member 2	-2.0
MGA	MGA, MAX dimerization protein	-2.0
UBA1	Ubiquitin like modifier activating enzyme 1	-2.0
POTEF	POTE ankyrin domain family member F	-2.0
SDPR	Caveolae associated protein 2(cavin2)	-2.0
SVIL	Supervillin	-2.0
TAF1L	TATA-box binding protein associated factor 1 like	-2.0
C5orf17	Chromosome 5 open reading frame 17	-1.9
TFAMP1	Transcription factor A, mitochondrial pseudogene 1	-1.9
EWSR1	EWS RNA binding protein 1	-1.9
CPD	Carboxypeptidase D	-1.9

ANP32D	Acidic nuclear phosphoprotein 32 family member	-1.9
LOC374443	C-type lectin domain family 2 member D pseudogene	-1.9
KIF4B	Kinesin family member 4B	-1.9
ZNF106	Zinc finger protein 106	-1.9
FLG	Fillagrin	-1.9
ZFHX3	Zinc finger homeobox 3	-1.8
ZNF138	Zinc finger protein 138	-1.8
LOC100288842	UDP-glcnac:betagal beta-1,3-N-acetylglucosaminyltransferase 5 pseudogene	-1.8
E2F3	E2F transcription factor 3	-1.8
HERC5	HECT and RLD domain containing E3 ubiquitin protein ligase 5	-1.8
TMEM5-AS1	TMEM5 antisense RNA 1	-1.8
NAP1L2	Nucleosome assembly protein 1 like 2	-1.8
XRCC6P5	X-ray repair cross complementing 6 pseudogene	-1.8
KPNB1	Karyopherin subunit beta 1	-1.8
DPYSL3	Dihydropyrimidinase like 3	-1.8
NEAT1	Nuclear paraspeckle assembly transcript 1 (non-protein coding)	-1.8
AURKAPS1	Aurora kinase A pseudogene 1	-1.7
ANKRD36BP1	Ankyrin repeat domain 36B pseudogene 1	-1.7
KIAA0754	Kiaa0754	-1.7
METTL7A	Methyltransferase like 7A	-1.7
SAMHD1	SAM and HD domain containing deoxynucleoside triphosphate triphosphohydrolase 1	-1.7
PIK3R1	Phosphoinositide-3-kinase regulatory subunit 1	-1.7
PRPS1L1	Phosphoribosyl pyrophosphate synthetase 1-like 1	-1.7
STAG3L2	Stromal antigen 3-like 2 (pseudogene)	-1.7
MMP13	Matrix metallopeptidase 13	-1.7
ASPM	Abnormal spindle microtubule assembly	-1.7
CHD8	Chromodomain helicase DNA binding protein 8	-1.7
SFT2D2	SFT2 domain containing 2	-1.7
FAM72C	Family with sequence similarity 72 member C	-1.7

DDX21	Dexd-box helicase 21	-1.6
CCDC144B	Coiled-coil domain containing 144B (pseudogene)	-1.6
NCR3LG1	Natural killer cell cytotoxicity receptor 3 ligand 1	-1.6
HELZ2	Helicase with zinc finger 2	-1.6
RSAD2	Radical S-adenosyl methionine domain containing 2	-1.6
BMS1P6	BMS1, ribosome biogenesis factor pseudogene 6	-1.6
ANAPC5	Anaphase promoting complex subunit 5	-1.6
JRK	Jrk helix-turn-helix protein	-1.6
USP1	Ubiquitin specific peptidase 1	-1.6
TRANK1	Tetratricopeptide repeat and ankyrin repeat containing 1	-1.6
NEU3	Neuraminidase 3	-1.6
OASL	2'-5'-oligoadenylate synthetase like	-1.6
SLC25A24	Solute carrier family 25 member 24	-1.5
GBP4	Guanylate binding protein 4	-1.5
CSMD3	CUB and Sushi multiple domains 3	-1.5
MFN2	Mitofusin 2	-1.5
MUC4	Mucin 4, cell surface associated	-1.5
POLR1A	RNA polymerase I subunit A	-1.5
TRPV6	Transient receptor potential cation channel subfamily V member 6	-1.5
UBA5	Ubiquitin like modifier activating enzyme 5	-1.5
TRIM66	Tripartite motif containing 66	-1.5
LOC494127	NFYC pseudogene	-1.5
LOC728024	Chromosome X open reading frame 56 pseudogene	-1.4
MIA3	MIA family member 3, ER export factor	-1.4
LOC401127	WD repeat domain 5 pseudogene	-1.4
LOC341056	SUMO1 activating enzyme subunit 1 pseudogene	-1.4
PANDAR	Promoter of CDKN1A antisense DNA damage activated RNA	-1.4
DCAF13P3	DDB1 and CUL4 associated factor 13 pseudogene 3	-1.4
