# Supplementary material for: Dual RNA-Seq Analysis of Trichophyton rubrum and HaCat Keratinocyte Co-Culture Highlights Important Genes for Fungal-Host Interaction
Source: Genes (Basel). 2018 Jul 19;9(7):362. doi: 10.3390/genes9070362 (PMC6070946; doi:10.3390/genes9070362)
Supplement: Supplementary file 1 [file genes-09-00362-s001.zip › Supplementary Table S3.pdf]

Supplementary Table S3. Complete list of genes differentially expressed in *T. rubrum* after 24 h of co-culture.

ID	Gene product name	Log_2 fold change
TERG_12606	Dipeptidylpeptidase V (DPPV)	2.2
TERG_01280	Hypothetical protein	2.1
TERG_03102	Sterol 24-C-methyltransferase- ERG6	2.1
TERG_08104	Potassium/sodium efflux P-type ATPase	2.0
TERG_01281	Malate synthase	1.7
TERG_04399	Phthalate transporter	1.6
TERG_00348	Galactose-proton symporter	1.4
TERG_02811	Hypothetical protein	1.4
TERG_12645	Hypothetical protein	1.3
TERG_07017	Oxidoreductase	1.3
TERG_08333	1-pyrroline-5-carboxylate dehydrogenase	1.3
TERG_02671	Hypothetical protein	1.3
TERG_02023	Extracellular matrix protein	1.3
TERG_08405	Leucine aminopeptidase 2	1.3
TERG_00916	Carboxylic acid transporter	1.3
TERG_11638	Isocitrate lyase	1.3
TERG_04952	ABC transporter	1.2
TERG_08286	Hypothetical protein	1.2
TERG_03712	Hypothetical protein	1.1
TERG_11639	Isocitrate lyase	1.1
TERG_01270	AMP dependent ligase	1.1
TERG_07691	Nonspecific lipid-transfer protein	1.1
TERG_07783	Oligopeptide transporter	1.1
TERG_02186	ABC transporter	1.1
TERG_04356	Amino acid permease	1.1
TERG_04580	NADP-specific glutamate dehydrogenase	1.0

TERG_01406	Hypothetical protein	-2.9
TERG_07726	Hypothetical protein	-2.2
TERG_03174	MFS siderochrome iron transporter	-2.0
TERG_06355	Hypothetical protein	-1.9
TERG_07035	Hypothetical protein	-1.8
TERG_04156	Hypothetical protein	-1.8
TERG_05655	AN1 zinc finger protein	-1.7
TERG_01622	Hypothetical protein	-1.6
TERG_07477	Hypothetical protein	-1.6
TERG_06186	Protein disulfide-isomerase domain-containing protein	-1.6
TERG_03708	Hypothetical protein	-1.5
TERG_03855	Hypothetical protein	-1.5
TERG_00499	Hypothetical protein	-1.4
TERG_03175	Hypothetical protein	-1.4
TERG_04073	Glutathione synthetase	-1.4
TERG_125	Hypothetical protein	-1.4
TERG_08139	NAD dependent epimerase/dehydratase	-1.3
TERG_06963	Hsp90-like protein	-1.3
TERG_01731	Hypothetical protein	-1.3
TERG_03340	Oxidoreductase	-1.3
TERG_01659	Hypothetical protein	-1.3
TERG_04863	Hypothetical protein	-1.3
TERG_01988	Hypothetical protein	-1.2
TERG_12510	Hypothetical protein	-1.2
TERG_07949	Heat shock protein 78	-1.2
TERG_04043	Hypothetical protein	-1.2
TERG_11780	Hypothetical protein	-1.2
TERG_00175	Hypothetical protein	-1.2
TERG_02278	Hypothetical protein	-1.2

TERG_05575	MFS multidrug transporter	-1.1
TERG_06315	Hypothetical protein	-1.1
TERG_07083	Hypothetical protein	-1.1
TERG_03070	Hypothetical protein	-1.0
TERG_07364	Hypothetical protein	-1.0
TERG_04966	Hypothetical protein	-1.0
TERG_00070	Maleylacetoacetate isomerase	-1.0
