# Supplementary figures and images for: Dual RNA-Seq Analysis of Trichophyton rubrum and HaCat Keratinocyte Co-Culture Highlights Important Genes for Fungal-Host Interaction
Source: Genes (Basel). 2018 Jul 19;9(7):362. doi: 10.3390/genes9070362 (PMC6070946; doi:10.3390/genes9070362)

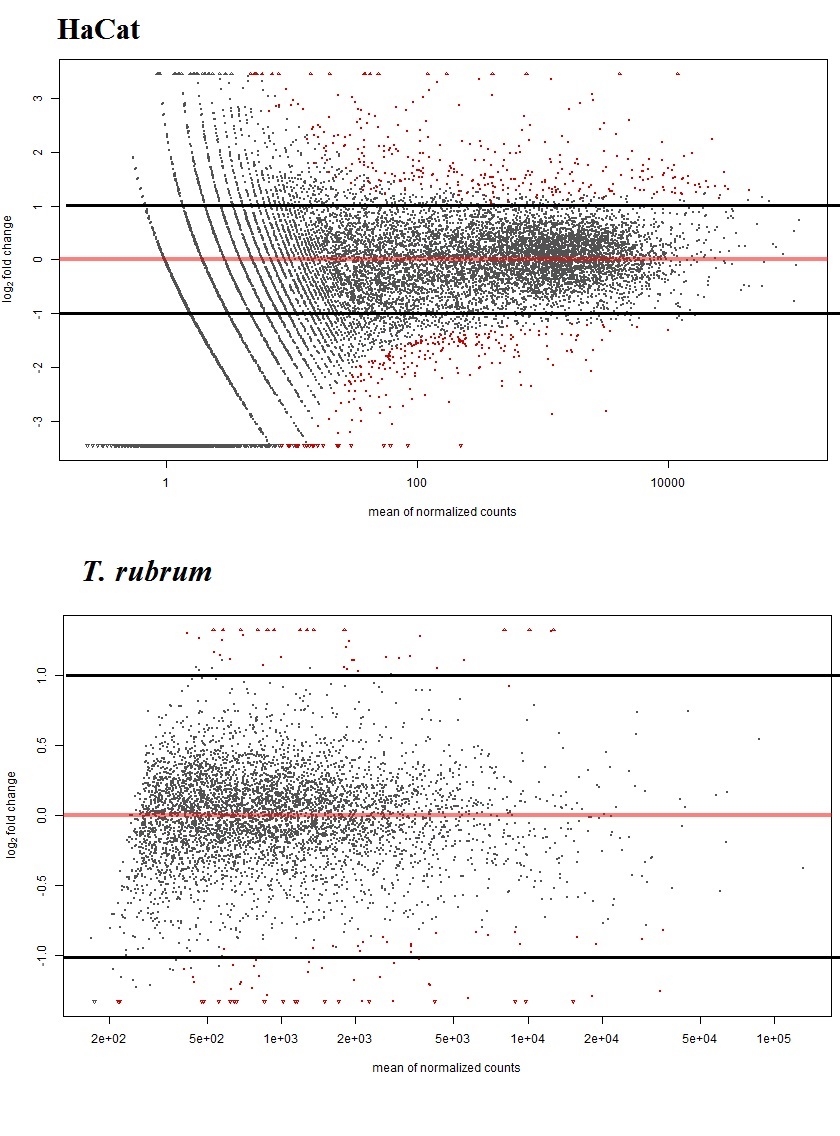

Supplement: Supplementary file 1 [file genes-09-00362-s001.zip › Supplementary Figure S1.jpg]
